# CONCORD biomarker prediction for novel drug introduction to different cancer types

**DOI:** 10.18632/oncotarget.23124

**Published:** 2017-12-09

**Authors:** Youngchul Kim, Patrick M. Dillon, Taesung Park, Jae K. Lee

**Affiliations:** ^1^ Department of Biostatistics and Bioinformatics, Moffitt Cancer Center, Tampa, FL, USA; ^2^ Division of Hematology/Oncology, University of Virginia, Charlottesville, VA, USA; ^3^ Department of Statistics, Seoul National University, Seoul, Korea

**Keywords:** gene expression model, drug repositioning, anticancer compound screening, cancer cell line, anti-cancer drug response prediction

## Abstract

Many cancer therapeutic agents have shown to be effective for treating multiple cancer types. Yet major challenges exist toward introducing a novel drug used in one cancer type to different cancer types, especially when a relatively small number of patients with the other cancer type often benefit from anti-cancer therapy with the drug. Recently, many novel agents were introduced to different cancer types together with companion biomarkers which were obtained or biologically assumed from the original cancer type. However, there is no guarantee that biomarkers from one cancer can directly predict a therapeutic response in another. To tackle this challenging question, we have developed a concordant expression biomarker-based technique (“CONCORD”) that overcomes these limitations. CONCORD predicts drug responses from one cancer type to another by identifying concordantly co-expressed biomarkers across different cancer systems. Application of CONCORD to three standard chemotherapeutic agents and two targeted agents demonstrated its ability to accurately predict the effectiveness of a drug against new cancer types and predict therapeutic response in patients.

## INTRODUCTION

Cancers harbor genetic aberrations and epigenetic changes that lead to considerable heterogeneity among patients with the same malignancy [1]. Accordingly, their response to a specific treatment can vary, depending on a variety of factors. Example includes tumor-specific factors (e.g. subtype, stage, genotype/phenotype, and heterogeneity) and patient-specific factors (e.g. pre-existing conditions and environment). Oncologic drugs currently do not have regulatory approval to be used across a broad range of cancer subtypes, due to historic developments and regulatory requirements of validation studies in a cancer body site rather than in a cancer genotype. Nevertheless, we now know that both cytotoxic and targeted drugs can work across different cancer sites and subtypes. Even some targeted small molecule inhibitors have unexplained activity in tumors that lack the target biomarker. These observations—combined with the myriad of novel cytotoxic and targeted drugs in the pipeline—indicate an urgent need to develop strategies for understanding how tumors respond to drugs more comprehensively than traditional single-site studies [2]. In fact, introducing novel drugs used in one cancer type to another has been one of the most successful strategies to date [3–5]. However, the practice of introducing drugs to different types of cancer is mostly performed on a trial-and-error basis (instead of being guided by objective metrics such as biomarkers) or rely on markers only from the original cancer type, which may not be relevant [6–9]. Consequently, even if a novel drug may show efficacy across different sites of cancer origin for subsets of patients, these results are often not applicable to the broader population [10, 11]. Ideally, the provision of anticancer therapies across various tumor types would be guided by an objective, biomarker-based model. This model would incorporate data from the original malignancy to identify accurate therapeutic enrichment biomarkers for other cancer types. Such a tool would significantly improve the success rates of introducing a new drug to different cancer types [12, 13].

Recent studies have demonstrated the ability to predict responses to anticancer therapies based on molecular biomarker signatures [14–21]. While most of these studies are still limited to predictions within single cancer type, a large number of cancer data sets are now available for examining biomarkers for the same therapeutic agents across different cancer types. This information potentially offers a strategy to identify concordant drug biomarkers across multiple malignancies [22, 23]. Here, we introduce a prediction methodology (“CONCORD**”**) designed to predict a drug’s therapeutic efficacy across different cancer types based on concordantly coexpressed markers. We obtained and validated CONCORD expression signatures on more than 20 patient cohorts across a diverse range of cancer types, for which we simultaneously predicted a particular drug’s effectiveness in (i) patients with original cancer and (ii) those with different cancer types. By applying CONCORD to standard chemotherapy and targeted therapies used across multiple malignancies, this work highlights the potential of our approach to objectively inform the implementation of existing anticancer therapies in novel and effective ways.

## RESULTS

### Pipeline of CONCORD biomarker development

**Figure 1:**
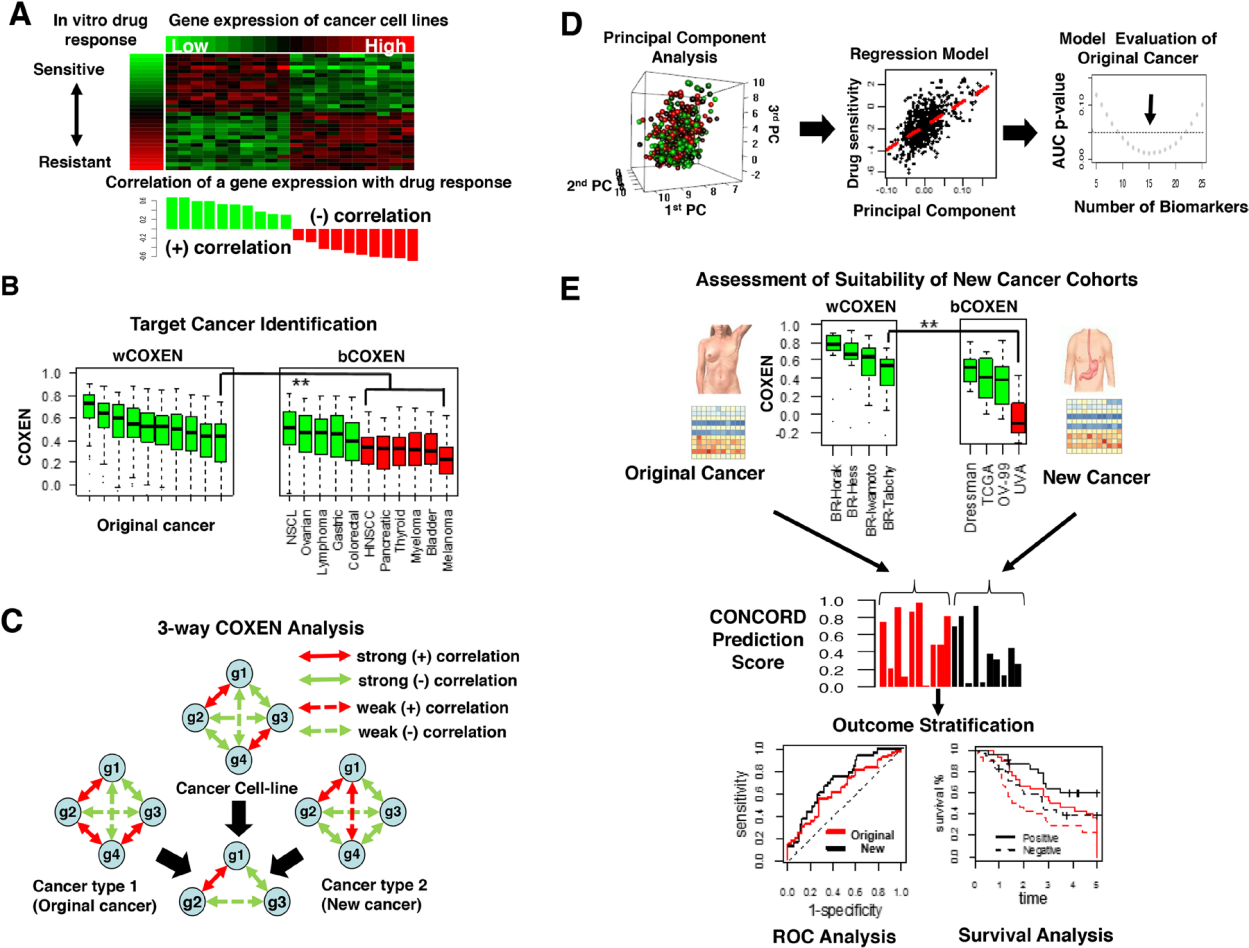
Overview of CONCORD drug response prediction **(A)** A heatmap of gene expression subset of NCI-60 cancer cell line panel and *in vitro* drug response profile, log GI-50, attached on the left side (top) and a bar graph showed positive (green) or negative correlation (red) between each gene expression and log GI-50 (bottom). **(B)** wCOXEN (left) and bCOXEN distributions (right): green boxes on bCOXEN indicate new cancer types suitable for cross-cancer type prediction from an original cancer. **(C)** A conceptual example of 3-way COXEN analysis: 4 genes resulted in 3 concordantly co-expressed genes across cell line and two human cancer subtypes. Solid and dotted line arrows indicate strong and weak correlation coefficients between connected genes, and red and green colored arrows represent positive and negative correlations, respectively. **(D)** Principal components of gene expression of drug sensitivity genes (left), multiple linear regression of GI-50 values on the principal components (middle), selecting a final predictor though testing performances of multiple candidate models (right). **(E)** Assessment of suitability of cohorts in new cancer types for cross-cancer prediction and validation (top), distribution of CONCORD prediction scores in original cancer (red) and new cancer cohort (black) (middle), comparisons of ROC or survival curves for external validation of cross-cancer type prediction (bottom).

**Figure 2:**
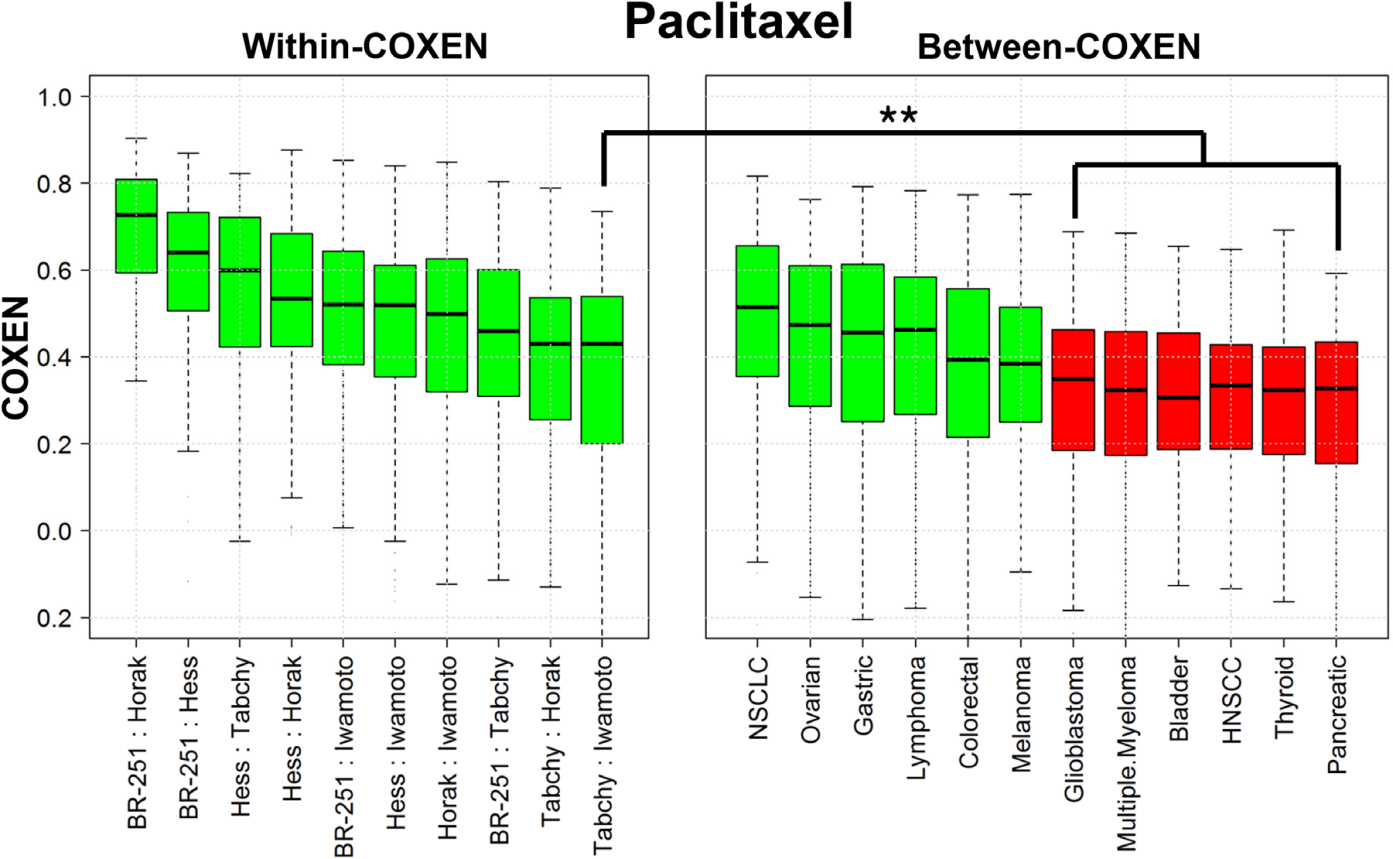
wCOXEN and bCOXEN of paclitaxel chemo-sensitivity biomarkers Distribution of COXENs within breast cancer (wCOXEN; left panel) and that of COXEN between breast cancer and each of other cancer types (bCOXEN; right panel). bCOXEN boxplots were sorted by Wilcoxon rank sum test p-values. Green bCOXEN boxplots represent cancer types relevant to cross-cancer prediction by having statistically insignificant difference from the lowest wCOXEN (^**^ Bonferroni-adjusted Wilcoxon rank sum test p-value < 0.025).

**Figure 3:**
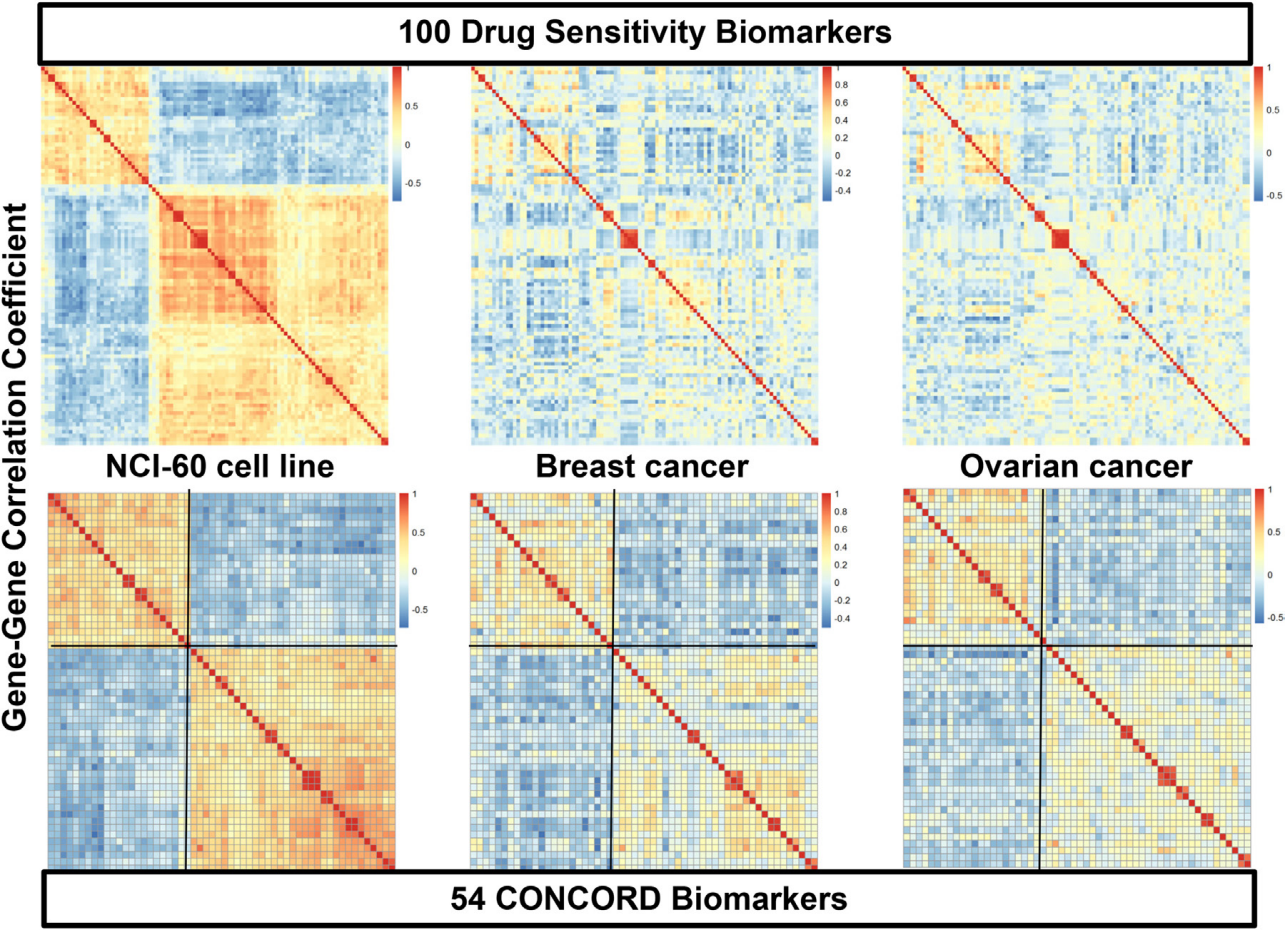
Heatmaps of gene-gene correlation matrices of initial drug sensitivity biomarkers and CONCORD biomarkers The top three clustering heatmaps display patterns of gene-gene correlations of the top 100 biomarkers of paclitaxel in three different cancer systems, NCI-60 cancer cell lines (top left), breast cancer (BR-251) (top middle), and ovarian cancer (OV-99) cohorts (top bottom). The bottom three panels show gene-gene correlation patterns of 54 concordantly co-expressed genes in the three-way COXEN analysis. Genes in breast and ovarian cancer heatmaps were sorted by the same order of genes in hierarchical cluster of NCI-60 correlation matrix.

**Figure 4:**
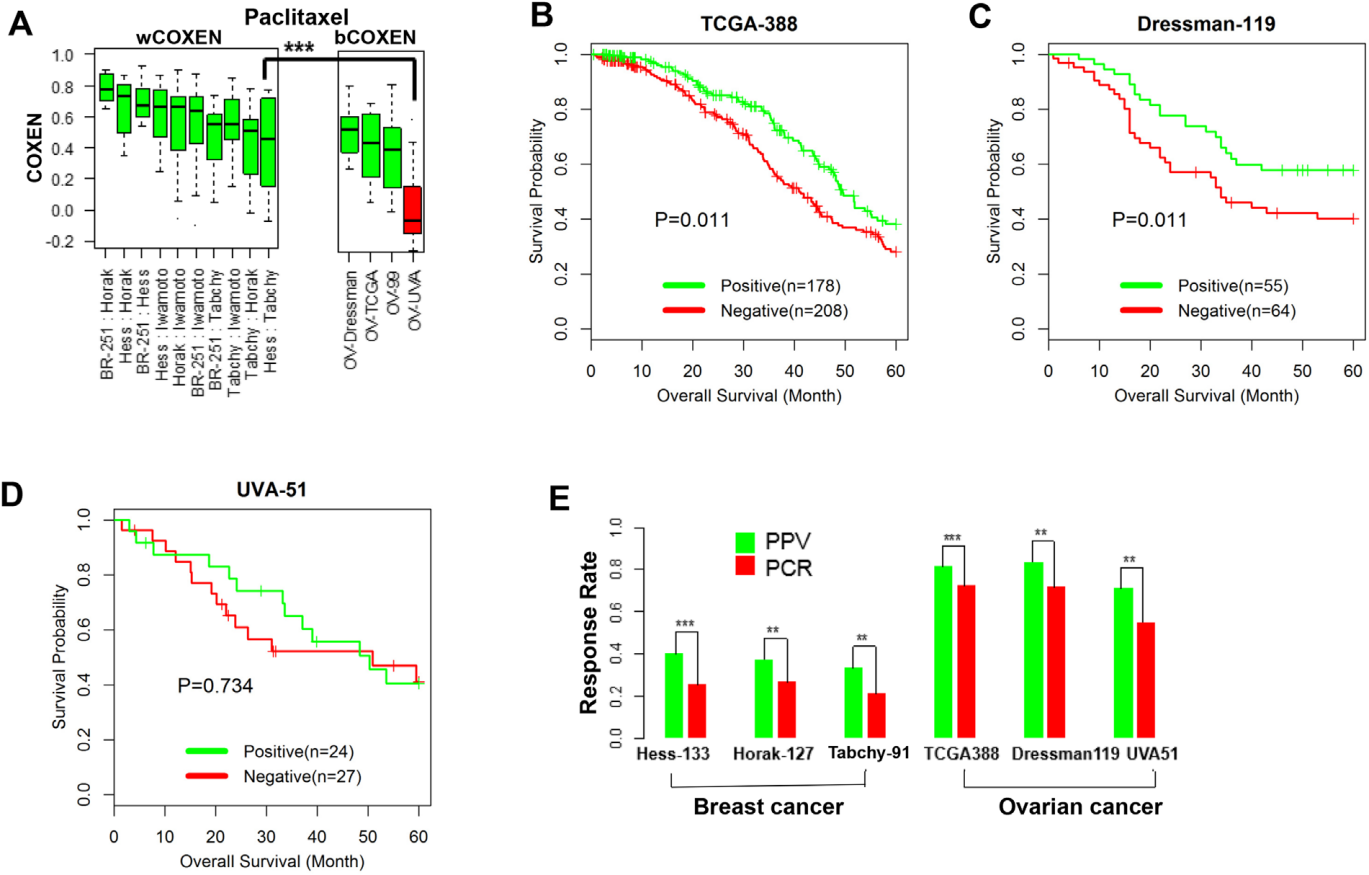
Cross-cancer type prediction of response to paclitaxel **(A)** wCOXEN and bCOXEN of final paclitaxel biomarkers in breast cancer and ovarian cancer cohorts: a red boxplot represents an ovarian cancer cohort, UVA-51, with significantly poor bCOXEN distribution, indicating unfitted for cross-cancer prediction. **(B)** Kaplan-Meier (KM) survival curves were calculated in predicted-positive (responsive) and predicted-negative (nonresponsive) groups of patients after outcome stratification in ovarian cancer cohorts and there are significant survival difference between the two groups in TCGA-388 (log rank-test p=0.011) and **(C)** Dressman-199 cohort (p=0.041) **(D)** but not in UVA-51 cohort (p=0.734) **(E)** positive predictive value of paclitaxel predictor (green) and pathological complete response rate (red) in breast cancer and ovarian cancer cohorts (^**^ p<0.05, ^***^p < 0.01).

**Figure 5:**
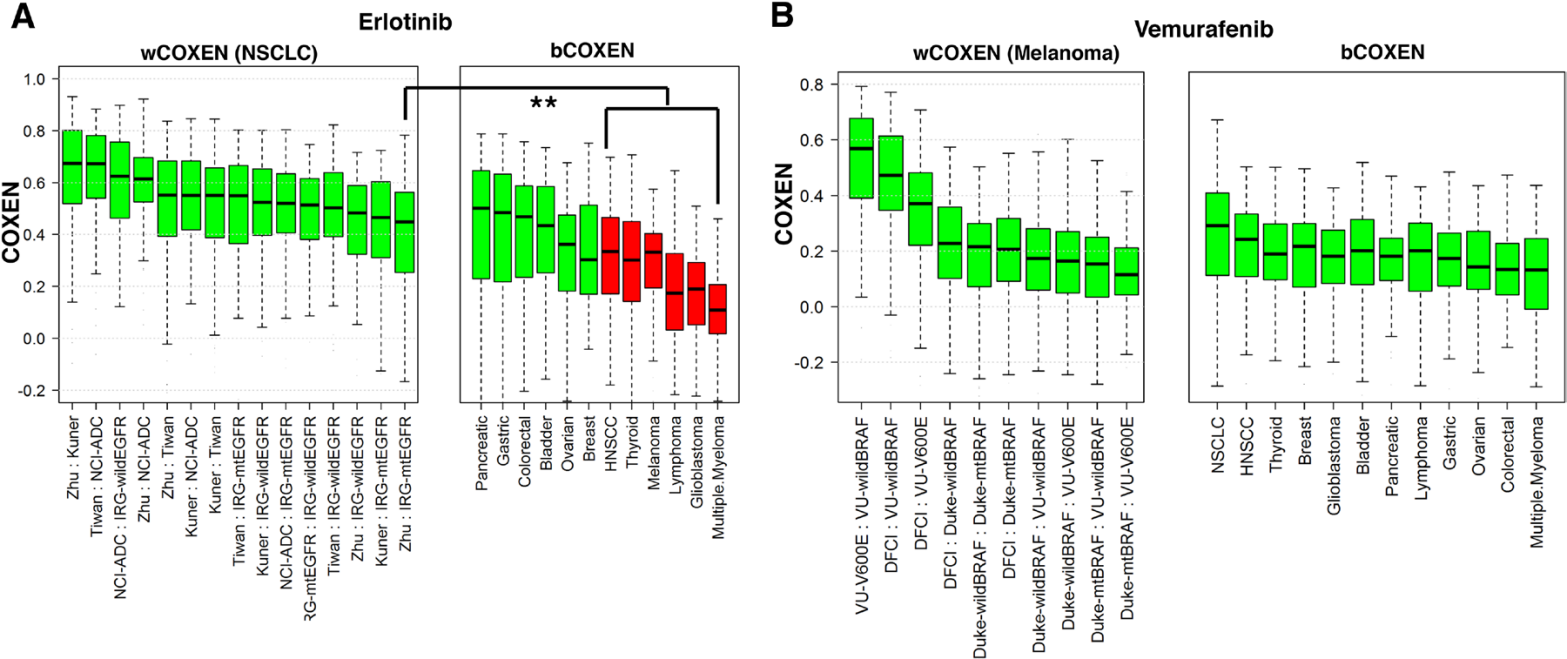
wCOXEN and bCOXEN of erlotinib and vemurafenib biomarkers **(A)** wCOXEN of 96 erlotinib sensitivity biomarkers in 6 NSCLC cohorts (left) and bCOXEN between NSCLC with mutant EGFR (IRG-mtEGFR) and each of twelve different types of cancer **(B)** wCOXEN of 125 vemurafenib sensitivity biomarkers in 5 melanoma cohorts (left panel) and bCOXEN between melanoma cohort with BRAF V600E mutation (VU-V600E) and other types of cancer (right panel) (^**^ p<0.05).

Our methodology consisted of six sequential steps: 1) discovery of *initial* drug sensitivity biomarkers on diverse cancer cell lines (Figure 1A), 2) identification of different cancer types that can be concordantly informed by the original cancer type (Figure 1B), 3) selection of three-way CONCORD biomarkers co-expressed across three inputs: cultured cancer cells, an original cancer, and new cancer sites (Figure 1C), 4) using gene expression of CONCORD biomarkers and drug activity data *in vitro* to train multigene expression models in the original cancer (Figure 1D), 5) validation on independent patient cohorts in the original cancer type (Figure 1E; top panel), and 6) prospective prediction of a patient’s response to the drug in other cancer types (Figure 1E; middle and bottom panels). A detailed description of the CONCORD algorithm is summarized in *Materials and Methods*.

We assessed the predictive ability of CONCORD for three cytotoxic chemotherapeutic agents used in multiple cancer types: Paclitaxel, 5-FU, and adriamycin. This approach allowed us to compare the predictive performance of CONCORD on outcomes and survival for these drugs in patients harboring the original cancer type versus new cancer types. CONCORD was also applied to two targeted kinase inhibitors, erlotinib and vermurafenib, to explore its applicability for targeted therapies. For instance, using paclitaxel, in step 1 we derived a set of drug sensitivity biomarkers from NCI-60 cancer cell line panel for nine different cancer types. In step 2, the original cancer type was breast cancer, for which paclitaxel is a standard chemotherapeutic agent. In step 3, the new cancer site was ovarian cancer, which we deemed suitable for cross-cancer prediction from breast cancer through an examination of within-COXEN (wCOXEN) and between-COXEN (bCOXEN) statistics in step 2. In step 4, principal component-based regression models were built on the basis of gene expression and paclitaxel activity profiles of the NCI-60 and tested on the largest breast cancer cohort, Hess-133, treated with paclitaxel. In steps 5 and 6, an optimal prediction model was validated on external breast cancer and ovarian cancer cohorts that were completely independent of the previous steps.

### Discovery of *in vitro* drug sensitivity biomarkers

In order to discover an initial set of biomarkers that predict response to a single therapeutic agent, we examined gene expression and pharmacological drug activity data of NCI-60, GDSC, and CCLE cancer cell lines [22–24]. When drug activity data was available on multiple cancer cell line panels, we chose a cancer cell line panel which yielded the largest number of genes whose expression levels were significantly associated with the drug activity data. While each cancer cell line panel was subject to its specific experimental conditions, the rationale for this cancer cell line panel selection strategy was based on the ability to statistically show the richest information for each drug. For paclitaxel, NCI-60 cancer cell line panel was found to be most informative. We identified 202 probe sets with significantly differential expressions between sensitive and resistant cell lines by the two-sample *t*-test under false discovery rate (FDR)<0.05. In comparison, only four and two significant probe sets were identified from GDSC and CCLE, respectively. Similarly, CCLE was the most informative cancer cell line panel for erlotinib response prediction with 96 differentially expressed probe sets while the NCI-60 and GDSC yielded 83 and 55 probe sets, respectively. For adriamycin, drug activity data were available on the GDSC and NCI-60. 499 probe sets had significantly differential expressions in GDSC versus only two in NCI-60. For 5-FU and vemurafenib, drug activity data existed only on NCI-60, for which we identified 611 and 125 probe sets with significant correlations between gene expression and drug activity data, respectively (Supplementary Table 1).

### Identification of other cancer types for CONCORD prediction

We compared wCOXENs within breast cancer and bCOXENs between breast cancer and different cancer types for the three chemotherapy drugs. For instance, the medians of bCOXENs were 0.514, 0.474, 0.463, 0.456, 0.394, and 0.384 with non-small cell lung (NSCLC), ovarian, lymphoma, gastric, colorectal cancer, and melanoma for paclitaxel biomarkers, which showed no significant difference from the lowest median wCOXEN within breast cancer (lowest median 0.43) (Figure 2). This implied that interactive gene expression patterns of this drug’s CONCORD biomarkers in the first six cancer types were concordant with those in breast cancer. Therefore, we expected that there would be reliable subsets of patients with these cancer types for which this drug’s CONCORD prediction model for breast cancer could be applicable. On the other hand, the bCOXENs between breast and other six cancer types such as glioblastoma (GBM), head and neck squamous cell carcinoma (HNSCC), and pancreatic cancer (PCC) were significantly lower than the wCOXENs within breast cancer. These were not expected applicable to this drug’s CONCORD model derived from breast cancer (Wilcoxon rank sum test p-value < 0.01). As for 5-FU, breast cancer showed high bCOXEN with NSCLC (median=0.548), lymphoma (0.481), gastric (0.47), and ovarian cancer (0.464) compared to the wCOXENs (lowest median 0.465) (Supplementary Figure 1). 5-FU is currently being used only in breast and gastric cancer treatment as standard therapies, but not in the other three cancer types. We thus performed our CONCORD analysis between breast and gastric cancer from available patient data. Similarly, the adriamycin bCOXENs were high with NSCLC (median=0.498), melanoma (0.469), thyroid cancer (0.414), lymphoma (0.412), and ovarian cancer (0.41) (Supplementary Figure 2). Adriamycin is known to be the most effective and most commonly used single cytostatic agent against thyroid carcinomas [25] and is still being used to treat lymphoma, ovarian, and NSCLC in combination with other drugs [26–28]. Among these, we were able to perform this drug’s CONCORD prediction for lymphoma since relevant data on gene expression and clinical outcomes were publicly available.

### Selection of three-way CONCORD biomarkers

To obtain each drug’s consistent CONCORD prediction model from one cancer type to another—as well as from cancer cell lines to human cancers—we further triaged the initial *in vitro* drug sensitivity biomarkers into those with concordant expression patterns across three cancer sets:cell line and two different human cancers. For this analysis we performed the COXEN analysis for three paired sets—cell line to the first cancer, cell line to the second cancer, and the first cancer to the second cancer. For example, 159 (78.7%) of 202 initial paclitaxel biomarkers showed highly concordant COXEN coefficients between NCI-60 and breast cancer, 177 (87.6%) biomarkers between NCI-60 and ovarian cancer, and 184 (91.1%) biomarkers between breast and ovarian cancer (Supplementary Table 1). Intersecting all three COXEN analyses, 142 biomarkers (70.3%) were found to be concordantly co-expressed among the three sets simultaneously. The effects of this three-way COXEN biomarker selection can be visualized well in a clustering analysis of gene-gene correlation matrix as shown in Figure 3. The clustering heatmaps of initial top 100 paclitaxel sensitivity biomarkers showed quite heterogeneous patterns among three cancer sets: cancer cell line panel, breast cancer, and ovarian cancer. However, clustering heatmaps of 54 CONCORD biomarkers showed considerably homogeneous patterns with two distinct clusters. Therefore, the selection of the CONCORD biomarkers helped us obtain drug sensitivity biomarkers that were more consistently expressed across all three cancer sets. Similarly, for 5-FU and adriamycin, 475 (77.7% of the initial biomarkers) and 377 (75.6%) CONCORD biomarkers were identified, respectively.

### Functional annotation of CONCORD biomarkers

Using the Ingenuity Pathway Analysis (IPA) tool (Qiagen, Inc., Redwood City), comprehensive functional annotation of CONCORD biomarkers was explored for their gene networks and biological functions known to be associated with human diseases. For paclitaxel we found that three genes were reported to be associated with ovarian cancer (HOXB2, MYC, and TFPI2; p=0.024) and two genes were strongly associated with tumorigenesis of breast cancer (DUSP2 and ITGA3; p<0.001) [29–31]. Also, five genes were identified in the gene pathways of estrogen-mediated S-phase and tumoricidal function. As for 5-FU biomarkers, 29 biomarkers were found to be involved in the cell cycle networks of RNA transcription. Additionally, several canonical pathways such as eIF2 signaling and cell cycle control of chromosomal replication were identified [32]. As for adriamycin biomarkers, 101 (29.4%) biomarkers were involved in cell death and survival. In particular, 4 genes (TP53, E2F1, NPM1, and SENP3) were identified in p14/p19ARF tumor suppression network (Full annotated predictor lists are available in Supplementary Table 2, 3, and 4) [33, 34].

### CONCORD biomarker*-based in vitro* multigene prediction modeling

Multiple competing multi-gene prediction models were built by gradually increasing the number of each drug’s CONCORD biomarkers by degree of drug sensitivity. These competing models were evaluated and compared for their ability to predict patient outcomes in the original cancer type. A threshold score of each CONCORD prediction model was then selected to classify future patients into responsive (positive) or nonresponsive (negative) patients to the drug by maximizing the Youden’s *J* index from ROC analysis. For paclitaxel, a final CONCORD prediction model with 16 biomarkers was selected from the evaluation against the Hess-133 breast cancer cohort. This provided AUC of 0.724 (Mann-Whitney-Wilcoxon test p<0.001) and threshold score of 0.541 for predicting a patient’s pathologic complete response (pCR) after a systematic neoadjuvant chemotherapy with this drug. Similarly, CONCORD prediction models were selected with 7 and 56 biomarkers for 5-FU and Adriamycin by evaluating the following two breast cancer cohorts treated with these drugs (respectively): Tabchy-178 and Horak-265. The lists of the final biomarkers of these drug predictors were provided in the Supplementary Table 5. ROC analysis also showed significant AUCs of 0.657 and 0.63 on these evaluation cohorts (Supplementary Figure 3).

### CONCORD prediction of ovarian cancer response to paclitaxel

The CONCORD prediction model of paclitaxel derived from breast cancer was used to stratify patients' drug responses and survival outcomes simultaneously for three ovarian cancer cohorts and two other independent breast cancer cohorts. We first compared wCOXENs (within breast cancer sets) with bCOXENs between breast and ovarian cancers (Figure 4A). Two ovarian cancer sets---Dressman-119 [35] and TCGA-388 [36] and two breast cancer sets-Horak-127 [37] and Tabchy-91 [14] showed concordant COXEN distributions by confirming that their bCOXENs were not statistically lower than wCOXENs within breast cancer. However, an ovarian cancer cohort, UVA-51 [38], whose microarray data were obtained from archived formalin-fixed paraffin-embedded (FFPE) tumor samples, showed a significantly lower bCOXEN with breast cancer. As the COXEN distributions were concordant, we then used the optimal cutoff derived from the original cancer type (breast cancer) to classify patient response in the second cancer type (ovarian cancer). That is, we stratified each of the three ovarian cancer cohorts into the predicted responder and non-responder groups at threshold value predefined in breast cancer. We found that overall survival of two ovarian cancer cohorts---TCGA-388 and Dressman-119 were significantly different between the predicted responder and non-responder groups at the threshold (Log rank test p-value=0.011 for TCGA-388; p=0.041 for Dressman-119) (Figure 4B and 4C). Therefore, the paclitaxel CONCORD predictor and threshold value which were derived from breast cancer were able to consistently stratify patient outcomes in ovarian cancer. We could not find a significant survival difference for the third ovarian cancer cohort UVA-51 at the same threshold, which showed a poor bCOXEN with breast cancer (Figure 4D). We also derived the positive predictive values (PPVs) at the predefined threshold for all the breast and ovarian cancer cohorts. PPVs were 81%, 83.6%, and 70.8% for the ovarian cancer cohorts TCGA-388, Dressman-119, and UVA-51, and 37.2% and 33.3% for the two independent breast cancer cohorts---Horak-127 and Tabchy-91, respectively (Figure 4E). Therefore, PPVs for the patients with the CONCORD prediction scores were significantly higher than the actual pCR rates in these studies, implying that an enrichment strategy based on the CONCORD prediction scores could result in significantly higher response rates for these patients. We also evaluated overall prediction performance of this predictor on the three ovarian cancer cohorts by an ROC analysis independent of the pre-specified threshold value derived from breast cancer. From this analysis we found that the CONCORD model was significantly predictive for all three cohorts, including UVA-51 (Table 1 and Supplementary Figure 4). Therefore, the paclitaxel CONCORD predictor retained an overall predictive power for all ovarian cancer sets. However, the breast cancer-derived threshold value did not provide significant stratification for patient’s overall survival when a patient set such as UVA-51 showed significantly lower bCOXEN distributions with breast cancer.

**Table 1:** Receiver operating characteristic curves of CONCORD prediction on independent validation cohorts

Drug	Cancer Type	Validation Cohort	AUC (SE)	P-value	95% Confidence Interval of AUC
**Paclitaxel**	**Breast**	Hess-133	0.724 (0.048)	< 0.001	[0.629, 0.818]
		Horak-127	0.663 (0.055)	0.005	[0.556, 0.77]
		Tabchy-91	0.681 (0.066)	0.016	[0.552, 0.81]
	**Ovarian**	TCGA-448	0.602 (0.035)	0.004	[0.534, 0.671]
		UVA-51	0.725 (0.072)	0.006	[0.584, 0.867]
		Dressman-119	0.647 (0.054)	0.013	[0.54, 0.753]
**5-FU**	**Breast**	Tabchy-178	0.626 (0.06)	0.041	[0.509, 0.743]
		Hess-133	0.620 (0.057)	0.037	[0.508, 0.733]
		Iwamoto-82	0.644 (0.066)	0.041	[0.514, 0.774]
	**Gastric**	Kim-97	0.671 (0.065)	0.015	[0.543, 0.799]
**Adriamycin**	**Breast**	Horak-265	0.605 (0.047)	0.035	[0.513, 0.696]
		Tabchy-178	0.628 (0.062)	0.038	[0.505, 0.75]
		Hess-133	0.654 (0.058)	0.008	[0.539, 0.768]
		Iwamoto-82	0.72 (0.061)	0.002	[0.6, 0.84]
	**Lymphoma**	Steidl-130	0.601 (0.052)	0.07	[0.5, 0.703]
		Hummel-110	0.666 (0.051)	0.003	[0.565, 0.767]

Sensitivity and specificity of CONCORD prediction scores for predicting complete response outcomes in patients of each validation cohort were used to calculate the area under a receiver operating characteristic curve (AUC), standard error (SE), and 95% confidence interval of AUC. P-value is a result of significance test of AUC against a null hypothesized AUC of 0.5, which means a random prediction.

### CONCORD prediction of gastric cancer response to 5-FU

Gastric cancer was identified as one of the top candidate cancer types for our CONCORD prediction derived from breast cancer response to 5-FU (Supplementary Figure 1). Therefore, a CONCORD model of 5-FU was obtained between breast and gastric cancers, and applied simultaneously to two independent breast cancer cohorts (Hess-133 [39] and Iwamoto-82 [40]) and one gastric cancer cohort (Kim-97 [41]) (Supplementary Figure 5A). Data on clinical response was available for these studies, but long-term survival information was unavailable. Thus our prediction validation was performed only for stratifying clinical response. For the gastric cancer patient cohort (Kim-97), the predictor provided the PPV of 91.2%, which was again significantly higher than its observed pCR rate of 77.3% (p<0.01) at the threshold value derived from breast cancer (Supplementary Figure 5B). In breast cancer PPVs were 37% and 41.4% for Hess-133 and Iwamoto-82, compared to their observed pCR rates 25.6% (p=0.022) and 29.3% (p=0.051), respectively. Thus, these results also showed that this 5-FU CONCORD model could be effectively used for predicting both breast and gastric cancer patient responses at the same threshold value of the drug predictor. The ROC analysis for overall prediction performance also showed that it performed well in all three cancer cohorts (Table 1 and Supplementary Figure 6).

### CONCORD prediction of adriamycin for lymphoma

From the initial COXEN analysis (Step 2), lymphoma was identified as one of the top candidate cancer types for predicting response to adriamycin based on the CONCORD model for breast cancer. Thus, we derived a CONCORD prediction model of adriamycin from breast cancer and applied it simultaneously to four lymphoma cohorts and three independent breast cancer cohorts. We found that one lymphoma set, Dave-24 [42], showed a relatively low bCOXEN with breast cancer (p<0.001, Supplementary Figure 7A). We confirmed that there were, at least, marginally significant differences in overall survival for Hummel-110 [43] (p=0.072) and Lenz-414 [44] (p=0.036). On the other hand, for Dave-24, which showed a significantly lower bCOXEN, the overall survival difference was not significant (p=0.817) (Supplementary Figure 8). When clinical responses of the two lymphoma cohorts were classified at the predefined threshold, PPVs were significantly higher with 81.6% for Steidl-130 [45] and 63.4% for Hummel-110 than their actual pCR rates 70.8% (p=0.019) and 49.1% (p=0.015), respectively (Supplementary Figure 7B). As for the breast cancer cohorts, PPVs were 38% for Hess-133 and 48.4% for Iwamoto-82; their pCR rates were 25.6% (p=0.014) and 29.3% (p=0.005). The Tabchy-178 cohort showed a marginally significant difference at the threshold (PPV=20.9%, pCR=14.6%, and p=0.06). However, ROC analysis provided significant AUCs for all breast cancer and lymphoma cohorts in predicting pCR, with the exception of Steidl-130, which showed a marginally significant result (AUC=0.601; p=0.07) (Table 1 and Supplementary Figure 9). Therefore, the CONCORD prediction model of adriamycin retained an overall predictive power for all lymphoma sets. However, patient stratification based on the threshold score predefined from breast cancer did not perform well when the bCOXEN was poor as compared to the wCOXEN between breast cancer patient sets.

### CONCORD prediction from a reverse direction

In the CONCORD applications above, we used breast cancer as the original cancer system because it allowed analysis on a relatively abundant set of patient data. However, we next sought to examine whether the “directionality” of our predictions was important. Specifically, since four independent cohorts of ovarian cancer patients having data on gene expression and pathological response to paclitaxel were available, we examined applicability of CONCORD for paclitaxel *from* ovarian cancer *to* breast cancer. It is worthwhile to note that for this reverse direction, most CONCORD analysis steps were identical, including initial drug sensitivity biomarker discovery on the cancer cell lines, three-way COXEN analysis on three cancer systems, and multigene prediction model training on the cell line panel. The major difference was the final CONCORD model selection step, which was based on the largest ovarian cancer cohort, TCGA-338. We found that the predictive performances for each direction were very similar. First, in the second step of CONCORD, breast cancer was uniquely selected with the highest bCOXEN (median=0.474) as the most relevant cancer type for a cross-cancer type prediction from ovarian cancer. This was after excluding wCOXEN of UVA-51, which used FFPE tumor tissue specimens and had consistently low wCOXENs with other ovarian cancer cohorts using frozen tumor tissues (Supplementary Figure 10a). In the CONCORD model selection step, multiple competing models were tested on TCGA-338. 16-CONCORD biomarkers model was very parsimonious and significantly predictive of pathological response (Supplementary Figure 10b). This model was exactly the same with the final model of CONCORD analysis from breast cancer because the same model training set and CONCORD biomarkers were used. The threshold value for patient stratification derived from TCGA-388 was 0.529. This value was almost identical to that of the breast cancer cohort (0.541). After stratifying patients into predicted responder and non-responder groups at the threshold value, significant survival differences were found in TCGA-388 and Dressman-119, but not in UVA-51. PPVs of prediction scores were consistently higher than pCR rates in all ovarian and breast cancer cohorts (Supplementary Figure 11).

### Application of CONCORD to targeted therapeutics

To explore cross-cancer predictive potential for targeted therapeutics, we applied CONCORD to two targeted therapeutic agents: Erlotinib (an epidermal growth factor receptor (EGFR) tyrosine kinase inhibitor) and Vemurafenib (a B-RAF inhibitor). These drugs are currently approved for treating advanced NSCLC with EGFR mutations and melanoma with B-RAF V600E mutation, respectively [46, 47]. For Erlotinib, we first derived 96 drug sensitivity biomarkers from the CCLE cancer cell line panel and used gene expression data of NSCLC patients’ tumors with mutant EGFR in the calculation of wCOXEN. Interestingly, three gastrointestinal cancer types including pancreatic, gastric, and colorectal cancer, and bladder cancer were then identified as the most promising cancer types for cross-cancer prediction from EGFR-mutant NSCLC by our CONCORD analysis (Figure 5A). As these targeted drugs have not been used in the other cancer types, no patient data sets were available for our direct validation of CONCORD predictions on these cancer types. However, we found that the efficacy of the drug has already been confirmed for these cancer types by several ongoing studies. Erlotinib has been approved for the advanced pancreatic cancer patients who have not received previous chemotherapy [48]. A phase 2 study of erlotinib in patients with metastatic colorectal cancer reported that more than one third of evaluable patients had stable disease with favorable toxicity profiles [49]. A recent randomized, open-label, phase 3 trial explored erlotinib plus Bevacizumab as a new non-chemotherapy-based maintenance option as a first line treatment for patients with unresectable metastatic colorectal cancer previously exposed to bevacizumab-based induction therapy [50]. Furthermore, a recent phase 2 study of bladder cancer reported that Erlotinib had beneficial effects in short-term clinical outcomes for patients with invasive bladder cancer [51]. As for vemurafenib, we derived 125 drug sensitivity biomarkers from NCI-60 cancer cell line panel and used gene expression of 19 melanoma patients bearing the target mutation of Vemurafenib (B-RAF V600E mutation) to calculate bCOXEN between melanoma and other cancer types. All cancer types evaluated showed high CONCORD cross-cancer predictive value for this drug from melanoma. NSCLC was the most plausible cancer type followed by HNSCC and breast cancers among others (Figure 5B). Again, no patient set treated with this drug was available for our direct validation with these selected cancer types. However, promising clinical efficacy of Vemurafenib has been reported in a recent phase 2 “basket” trial of vemurafenib in patients with non-melanoma cancers harboring BRAF V600E mutations. In the clinical trial, the second highest overall response rate of 42% was observed in NSCLC after 43% in Erdheim-Chester disease or Langerhans’-cell histiocytosis. Furthermore, there were anecdotal responses among patients with anaplastic thyroid cancer, ovarian cancer, and colorectal cancer [52]. Therefore, these studies suggest that CONCORD identification of cross-cancer types is consistent with current applications.

### Connectivity map application of CONCORD biomarkers with independent drug signatures

We also evaluated functional connections between drugs and their final CONCORD biomarkers by using the Connectivity Map (CMap), which has been widely used for drug repositioning analysis [11]. The CMap had reference profiles for paclitaxel and doxorubicin out of chemotherapy drugs. Thus, we queried our final sixteen paclitaxel biomarkers as a gene signature, which resulted in paclitaxel as top 107^th^ out of 6,100 instances (top 1.7 percentile) with a marginally significant connectivity score of -0.498 (p=0.067) and 66% non-null percentage (a measure of support for connection between a set of paclitaxel instances and input gene signature). In subsequent query of 50 final doxorubicin biomarkers, doxorubicin also ranked high at top 73^rd^ (1.2 percentile), with a statistically significant enrichment score of -0.772 and non-null percentage of 100% (p=0.024), implying that CONCORD gene signatures were associated with these drug activities (Supplementary Figure 12).

## DISCUSSION

We here introduced CONCORD, a novel biomarker prediction technique to forecast a drug’s therapeutic response for patients with a new cancer type for which the drug has not yet been used. We showed that the cross-cancer CONCORD prediction of several standard chemotherapy agents were significantly predictive for patient responses in different cancer types in an independent, prospective manner. In particular, when its bCOXEN was similar to wCOXEN in the original cancer type, we were able to directly use the predefined threshold from the original cancer to stratify patient outcomes in the second cancer type. We believe that there were several important components for our cross-cancer CONCORD prediction. First, we used *in vitro* cancer cell line panels consisting of diverse cancer types to discover each drug’s initial sensitivity biomarkers that could reflect its single drug effects across different cancer types. Second, we evaluated and compared the COXEN distributions to triage each drug’s biomarkers in a manner consistent with genetic co-expression patterns among three cancer systems—cell lines, patients with the initial cancer type and patients with the second cancer type. We found that such biomarkers could retain a concordant predictive power across different cancer types based on their consistent gene (co-expression) networks across the three cancer systems.

In our CONCORD applications to targeted therapeutics, several new cancer types such as pancreatic cancer and NSCLC were also identified as the highest potential cross-cancer types for Erlotinib and Vemurafenib. It was reported that overall response rate with BRAF therapy was 53% and disease control rate was 85% in BRAF-mutant lung cancer [53]. Hence, it will be interesting to examine novel opportunities to use these drugs on those cancer types by using CONCORD to predict patients with highest probabilities of responding.

There have been studies to effectively infer potential drug indications by either matching drug or disease gene expression profiles. The CMap is, for instance, a well-established systematic computational approach in which differential gene expression patterns were compared before and after each drug’s treatment on cancer cell lines. Our CONCORD has two distinct advantages over CMap. First, CONCORD is designed to provide not only new target cancer types for drug repositioning, but also an accurate statistical prediction model to select responsive patients with the new target cancer type. Second, CONCORD does not need any drug perturbation for its reference library construction (COXEN sets) and can use *de novo* gene expression data obtained before a drug’s treatment. The latter distinction will be highly important for introducing a novel drug in clinical settings. Also, CMap infers a novel agent’s pharmacological activities in its reference database but cannot directly select a new disease type for which a novel drug can be effective.

It is worth to note several limitations of our current study. Although we developed and validated several single-drug signatures, the majority of patients in the cancer cohorts used for this study were treated with multi-drug combinations such as T/FAC in breast cancer. It will therefore be very useful if inferences on single drug contributions can be made for patients who were treated with combination therapies. Yet this is challenging for several reasons: First, different chemotherapy drug effects are often correlated, so that it is difficult to decompose them solely into exclusive individual drug effects. Also, it is difficult to obtain and validate equally highly predictive biomarker models for all single drugs, and individual drug effects cannot be accurately estimated from their combination signature. Furthermore, single (and combination) drug effects are associated with many other confounding factors such as target patient population and specific clinical settings of each study. However, if high-performing single drug biomarker models for all drugs used in a specific combination regimen can be curated, this opens the possibility of using multivariate logistic regression models on the single drug signatures. This statistical model can then provide single drug effect coefficients, risk odds ratios, and p-values, which may provide, in part, information necessary for evaluating individual drug effects [21, 38]. These strategies are currently being investigated.

A tumor’s response and resistance to a therapeutic agent will rely not only on *de novo* pre-treatment cellular mechanisms but also on post-treatment molecular mechanisms and microenvironments after a selective pressure is applied. While our CONCORD attempted to utilize *de novo* molecular information for our cross-cancer prediction, the latter molecular information such as drug activities and resistance under certain cancer-specific mutations will not be apparent until the drug is actually used. Thus it will be important to obtain and integrate cancer-specific mutation and other molecular information to more accurately predict cross-cancer patient therapeutic responses.

We also found that it was difficult to discover consistent biomarkers from different RNA sources, (e.g. UVA-51 ovarian cancer cohort from FFPE tissue samples). It was important to overcome such technical differences by using appropriate quality control and normalization analysis procedures for our CONCORD prediction. In our current study, developments and validations were mainly applied to patient sets profiled with several Affymetrix microarray platforms. While some of our multi-gene biomarker models have been successfully applied with considerably different platforms, e.g., between oligo- and cDNA- microarrays by us and others, this technical limitation needs to be examined more carefully. [17, 20]. Also, our biomarker discovery and modelling methods are highly dependent on the cancer cell line panel and patient data resources in the initial cancer type; thus, our CONCORD approach is currently restricted to drugs and cancer types for which such rich datasets are available. It will be important to determine the minimum requirements for accurate CONCORD predictions in future studies.

It will also be useful to investigate whether CONCORD can be extended to different molecular data such as genome-wide mutations, aberrations, RNAseq, proteomics, or metabolomics data. We believe that the mathematical framework of CONCORD will be broadly applicable to these different molecular platforms. However, one may need to carefully examine if large reliable patient data resources are available and whether predictive therapeutic biomarkers can be obtained from such molecular data. Likewise, since cultured cell lines can show quite different expression profiles across many key genes, it may be possible to substitute cell line-based expression data from other sources such as patient-derived xenograft tumors, treated metastatic tumors, or other model systems (including *ex vivo* spheroid and autochthonous models). The ultimate utility of these CONCORD predictions should be assessed by a prospective study.

To share the CONCORD algorithm with the scientific community, a web-based CONCORD tool is currently under development. Using this tool, investigators can obtain drug sensitivity biomarkers for anti-cancer compounds screened in NCI-60, CCLE, and GDSC cancer cell line studies. The algorithm could also be applied to cross-cancer type drug response prediction using publicly-available gene expression data from Gene Expression Omnibus and ArrayExpress, along with users’ own gene expression data.

## MATERIALS AND METHODS

### Gene expression and drug sensitivity data of human cancer cell line panels

Microarray gene expression data sets of the NCI-60 cell line panel are available publicly at the National Cancer Institute (http://discover.nci.nih.gov/cellminer). *in vitro* drug sensitivity data, 50% growth inhibition (GI50), on the NCI-60 cell lines were obtained from the NCI DTP website (http://dtp.nci.nih.gov). The gene expression and drug sensitivity data of GDSC-648 cell line panel are available at the Welcome Trust Sanger Institute (http://www.cancerrxgene.org). The Cancer Cell Line Encyclopedia (CCLE) also provides public access for analyzing and visualizing gene expression and mutation data for over 1000 cancer cell lines encompassing 36 tumor types, and also pharmacological profiles for 24 anti-cancer drugs across 504 cell lines (http://www.broadinstitute.org/ccle).

### Cancer patient cohorts for CONCORD development and validation

The list of cancer cell line panels and cancer patient cohorts treated with corresponding cancer therapeutic agents is summarized in Supplementary Table 6 with its cancer types and roles in our CONCORD development. Patients in the breast cancer cohorts were treated with T/FAC (taxane / 5-FU, adriamycin and cyclophosphamide) or with FAC or FEC (5-FU, epirubicin, and cyclophophamide)[14, 39, 40]. Patients in all ovarian cancer cohorts were treated with platinum-based systematic chemotherapy with Taxane [35]. Non-Hodgkin lymphoma patients were treated with CHOP (cyclophosphamide, adriamycin, vincristine, and prednison) or R-CHOP (rituximab in addition to CHOP) and a Hodgkin lymphoma cohort, Steidel-130, was treated with AVCD (adriamycin, bleomycin, vinblastine, and dacarbazine) [42–44]. The COXEN sets were pretreatment gene expression microarray data for a large number of patients. These datasets were used to derive COXEN coefficients in cancer type selection and to identify three-way COXEN biomarkers. We have not used them for our drug sensitivity biomarker discovery and prediction modeling in any manner. In this study we used breast cancer as a primary cancer type since multiple large patient sets were available. These sets included important parameters such as pathologic clinical response after chemotherapy which was required for our independent model evaluation and optimal threshold derivation for cross-cancer patient stratification. Other cohorts of cancer patients treated with the drug of interest were used later for external validation of the final prediction model of drug response. All gene expression microarray data and drug activity profile data used in the study are publicly available in NCBI Gene Expression Omnibus (GEO), ArrayExpress, and The Cancer Genome Atlas (TCGA) official websites..

### Discovery of *in vitro* drug sensitivity biomarkers

We discovered expression of certain biomarkers that were significantly associated with each drug’s *in vitro* activities on cancer cell lines from multiple types of cancer. *In vitro* drug activity and gene expression data of cancer cell lines were used to screen the most accurate drug sensitivity biomarkers for a given drug. The basic unit of biomarker is an individual probe set in the microarray data of cancer cell lines. The drug sensitivity of each biomarker was then represented and prioritized by estimating either correlation coefficient of its gene expressions with drug sensitivity profiles or by independent two sample *t*-test statistics comparing gene expression levels between highly-sensitive and -resistant cell lines to the drug while controlling false discovery rate (FDR) at 0.05. These were ranked by strength of drug sensitivity in terms of absolute correlation-test or *t*-test statistics in a descending order. When activity data of a drug were available on multiple cancer cell lines panels, we chose a cancer cell line panel which yielded the largest number of significant gene probe sets in correlation and *t*-test analyses. We performed our statistical data analysis using the open-source statistical software R v3.1.0.

### Co-expression extrapolation (COXEN) coefficient

To quantify each probe’s co-expression relationships between two different cancer systems such as cell line panel and patient cohort (or two different cancer types), we calculated COXEN coefficient *rc(j)* for each probe *j* as follows. Using expression data within each of two systems separately, we constructed two correlation matrices (of dimension *n x n*) for *n* chemosensitivity biomarkers. The two correlation matrices, e.g. one for the NCI-60 and another for the cancer patient set such as BR-251, both were evaluated as *U = [U*_*ij*_*]*_*nxn*_ and *V = [V*_*ij*_*]*_*nxn*_, where *U*_*ij*_ and *V*_*ij*_ are the correlation coefficients between probes i and j in the NCI-60 and BR-251, respectively. Then, *rc(j)* is derived as

$$rc\left(j\right)=\frac{\sum_{k=1}^{n}\left(U_{kj}-{\bar{U}}_{j}\right)\left(V_{kj}-{\bar{V}}_{j}\right)}{\sqrt{\sum_{k=1}^{n}{\left(U_{kj}-{\bar{U}}_{j}\right)}^{2}}\sqrt{\sum_{k=1}^{n}{\left(V_{kj}-{\bar{V}}_{j}\right)}^{2}}}$$

where ${\bar{U}}_{j}$ and ${\bar{V}}_{j}$ are the mean correlation coefficients of the j-th column correlation coefficient vectors for the NCI-60 and BR-251. The COXEN coefficient *rc(j)* is thus simply the correlation coefficient between column vectors ${\bar{U}}_{j}$ and ${\bar{V}}_{j}$. Therefore, *rc(j)* reflects the degree of concordance between the NCI-60 and BR-251 panels for expression relationships of probe *j* with other *n-1* probes. If *rc(j)* exceeded a cut-off criterion e.g., 98^th^ percentile of the null distribution by random shuffling for two-sided test, probe j was selected as a significant COXEN probe between the two panels. Note that since probe *j* was initially selected among *n* chemosensitivity biomarkers, it also retained drug sensitivity characteristics.

### Identification of relevant cancer types for CONCORD prediction

We developed a method to identify new candidate cancer types for cross-cancer drug response prediction by comparing the COXEN distributions of predictive biomarkers of drug response within the same cancer type (wCOXEN; *within*-COXEN) with those between different types of cancer (bCOXEN; *between*-COXEN). Each of wCOXEN and bCOXEN is a set of COXEN coefficients of genes related to initial *in vitro* sensitivity to a drug. The former was calculated for any pairs of patient cohorts of a pre-specified original cancer type. The latter was calculated between the original cancer type cohort and a different cancer type cohort. To calculate bCOXEN, we defined the COXEN set for each cancer type as the largest patient cohort available with gene expression microarray data obtained before any cancer-related therapy. These COXEN sets were not used for our biomarker discovery and prediction modeling in any manner (see Supplementary Table 6). We then used a criterion that bCOXEN should be consistent with (or not significantly worse than) wCOXEN for our cross-cancer CONCORD prediction. To examine this criterion, we compared these bCOXENs to wCOXEN with the lowest median among gene expression data sets of the original cancer type by using one-sided Wilcoxon rank sum test at a significance level of 0.025. A null hypothesis for the test was that the median of bCOXEN is equivalent to the lowest median of wCOXEN. A corresponding alternative hypothesis was that the bCOXEN had a lower median than the lowest median of wCOXEN. Bonferroni-adjusted p-values were calculated to adjust for multiple comparisons of bCOXEN to wCOXEN. A different cancer type was considered suitable for the CONCORD prediction if its bCOXENs were statistically equivalent to wCOXENs (Wilcoxon rank sum test p-value>0.025).

### Three-way COXEN biomarker identification

Once a different cancer type was selected for CONCORD modeling, we further triaged the initial *in vitro* predictive biomarkers of drug response into the ones that retained highly significant COXEN coefficients across all the three pairs among cancer cell line, original cancer, and different cancer patient sets defined as COXEN sets in Supplementary Table 6. This step was intended to screen drug sensitivity biomarkers that were concordantly co-expressed across all three cancer systems—cell lines, original cancer site, and second cancer site. For this analysis we calculated COXEN coefficients for all initial drug sensitivity biomarkers and extracted biomarkers with significant COXEN under FDR < 0.2 in each of the pairs for three COXEN sets separately. For instance, COXEN coefficients of 202 paclitaxel drug sensitivity biomarkers were calculated for each of following three pairs; 1) the NCI-60 cancer cell line panel and BR-251 breast cancer cohort, 2) the NCI-60 and OV-99 ovarian cancer cohort, and finally 3) the BR-251 breast cancer and OV-99 ovarian cancer cohorts. We compared and intersected three sets of biomarkers with significant COXEN coefficients in each of the three pairs to identify significantly co-expressed common biomarkers across NCI-60, breast cancer, and ovarian cancer. Hence, the resultant biomarkers, which had significant COXEN coefficients across all three pairs, were classified as CONCORD biomarkers. This process can also be considered as a humanization step of the initial *in vitro* drug sensitivity biomarkers. Therefore, this was one of the key steps that enabled us to obtain the biomarkers to convey drug response information from one cancer type to another cancer type. For each drug’s biomarkers, pathway analysis was performed to investigate known genetic networks and gene functions by Ingenuity Pathway analysis (IPA; Ingenuity, Inc., Redwood City, CA).

### CONCORD modeling and evaluation

Multivariate prediction models for each drug’s response were built using gene expression data and drug response data of the cell line panel used in *in vitro* drug sensitivity biomarker discovery. We performed a principal component regression analysis using a statistical dimension reduction technique to avoid model overfitting due to a large number of biomarkers in the models. Competing models with different numbers of biomarkers were fitted from the most significant CONCORD biomarkers in stratifying *in vitro* drug sensitivity of cancer cell lines. To select the optimal prediction model for each drug, we independently evaluated the performance of competing models on the original cancer cohort with the largest number of patients treated with the drug. Each patient’s regression model prediction score was converted into a drug-sensitivity percentile score with one being the most sensitive and zero being the most resistant within the patient’s peer cohort. The performance of the optimal model was then evaluated by the receiver operating characteristic curve (ROC) with the area under the ROC curve (AUC) value. The best prediction model was then selected with most stable and significant prediction performance among competing models on the independent patient set of the original cancer type. For future response stratification (i.e., predicted responders vs. predicted non-responders), we also defined the threshold value for predicted positives vs. negatives by maximizing overall prediction performance with the Youden’s *J* index (=specificity + sensitivity -1) from the ROC analysis of the largest original cancer patient cohort. The final prediction model with the identical threshold value was used to stratify patients of all independent cohorts of both original and different cancer types, prospectively.

### CONCORD prediction and validation

We applied each drug’s final prediction model independently to historical patient cohorts of different cancer types. Beforehand, we evaluated the distribution of COXEN coefficients of final CONCORD biomarkers for each cohort of patients to assess its suitability to cross cancer prediction. The cohort with significantly lower bCOXEN than wCOXEN of the original cancer type was considered unsuitable for cross-cancer prediction. The performance of each drug’s CONCORD prediction was then assessed in a prospective manner. In addition to ROC and survival analyses, we also evaluated positive predictive value (PPV) based on the predefined threshold value from the original cancer type; thus, PPV would be a predicted rate of pCR with the CONCORD guided treatment. PPV was then compared with the observed pathological complete response rate (pCR) (under the current standard of care) for each cohort in which patients were unselectively treated. Therefore, this comparison would reflect the clinical benefits gained by the CONCORD-based patient enrichment. Statistical significance was obtained by a binomial proportion test for the difference between the expected PPV and observed pCR rate. For patient cohorts with survival outcome data, survival distributions were also compared between predicted positive and negative patient groups by Kaplan-Meier survival analysis with a log-rank test.

## SUPPLEMENTARY MATERIALS FIGURES AND TABLES















## Abbreviations

5-FU: Fluorouracil
AUC: area under a receiver operating characteristic curve
AVCD: Adriamycin, Bleomycin, Vinblastine, and Dacarbazine
CCLE: Cancer Cell Line Encyclopedia
GDSC: Genomics of Drug Sensitivity in Cancer
CHOP: Cyclophosphamide, Adriamycin, Vincristine, and Prednisone
COXEN: Co-Expression Extrapolation
wCOXEN: *within*-COXEN
bCOXEN: *between*-COXEN
FDR: false discovery rate
FFPE: formalin-fixed paraffin-embedded
IC-50: half-maximal inhibitory concentration
GI-50: 50% growth inhibition
GBM: glioblastoma multiforme
HNSCC: head and neck squamous cell carcinoma
NCI DTP: National Cancer Institute Developmental Therapeutics Program
NSCLC: non-small cell lung cancer
pCR: pathological complete response rate
PPV: positive predictive value
ROC: receiver operating characteristics
TCGA: The Cancer Genome Atlas
