# Supplementary material for: CONCORD biomarker prediction for novel drug introduction to different cancer types
Source: Oncotarget. 2017 Dec 9;9(1):1091–106. doi: 10.18632/oncotarget.23124 (PMC5787421; doi:10.18632/oncotarget.23124)
Supplement: Supplementary file 1 [file oncotarget-09-1091-s001.pdf]

CONCORD biomarker prediction for novel drug introduction to different cancer types

SUPPLEMENTARY MATERIALS

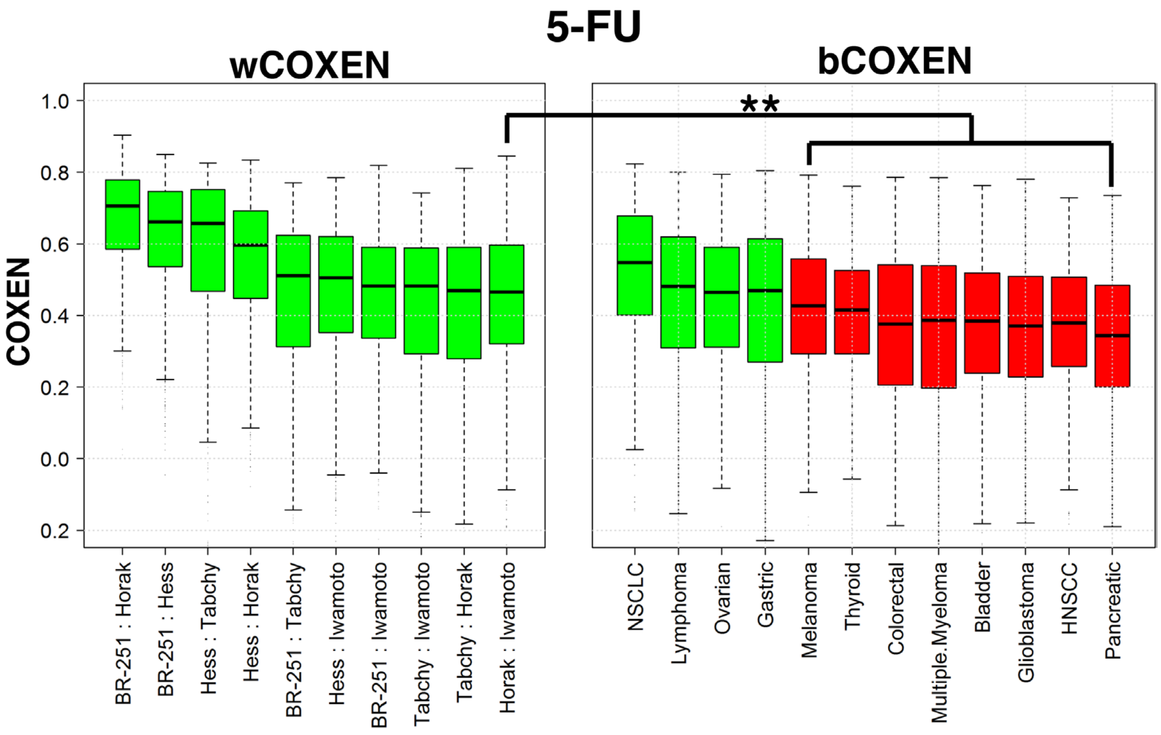

Supplementary Figure 1: 5-Fu: identification of cancer types for CONCORD prediction.

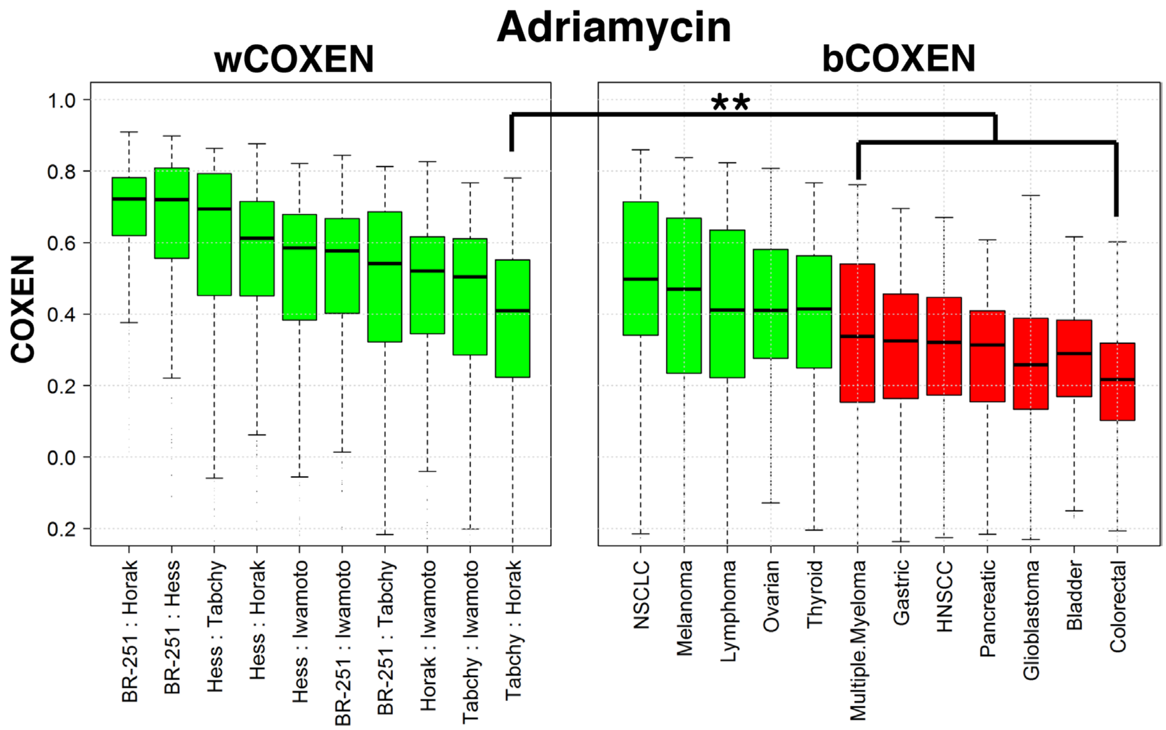

Supplementary Figure 2: Adriamycin: identification of cancer types for CONCORD prediction.

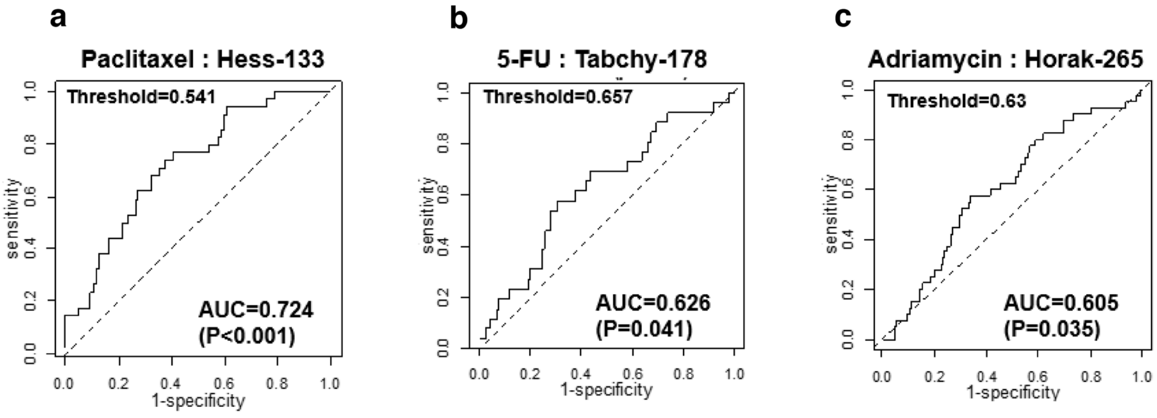

Supplementary Figure 3: ROC curves of final CONCORD models on breast cancer test sets.

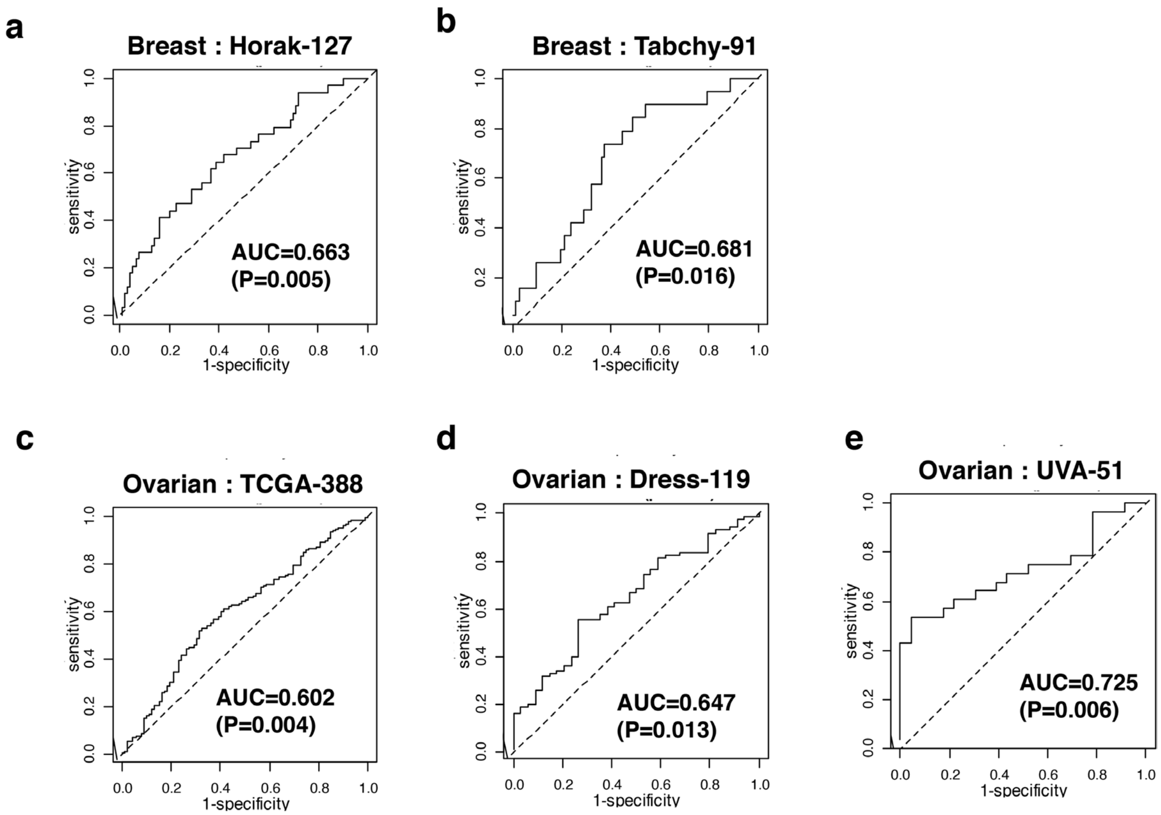

Supplementary Figure 4: Paclitaxel: ROC curves of CONCORD prediction on breast and ovarian cancers.

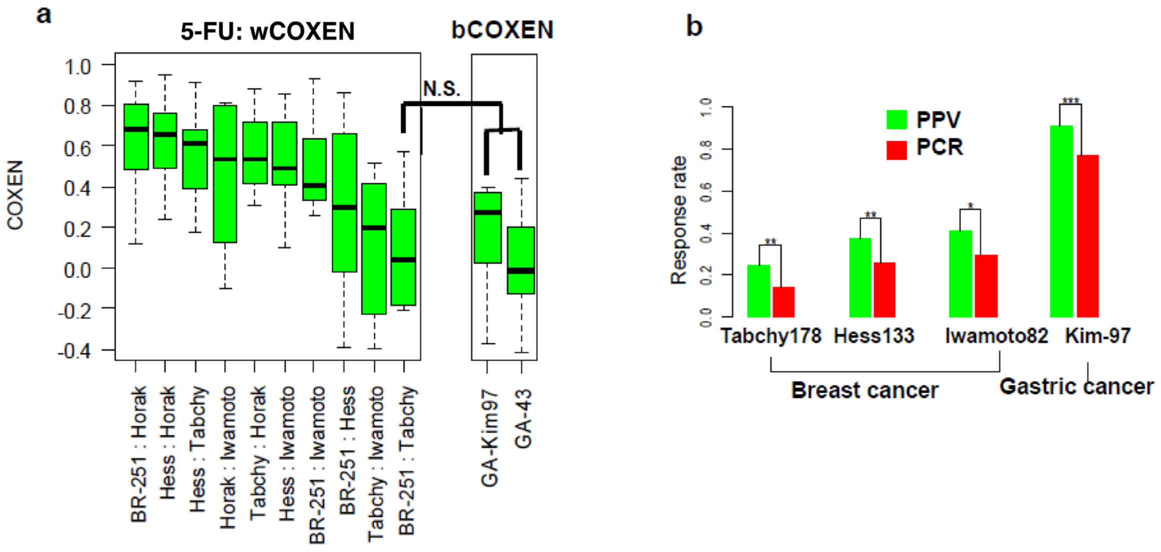

Supplementary Figure 5: 5-Fu: CONCORD cross-cancer prediction.

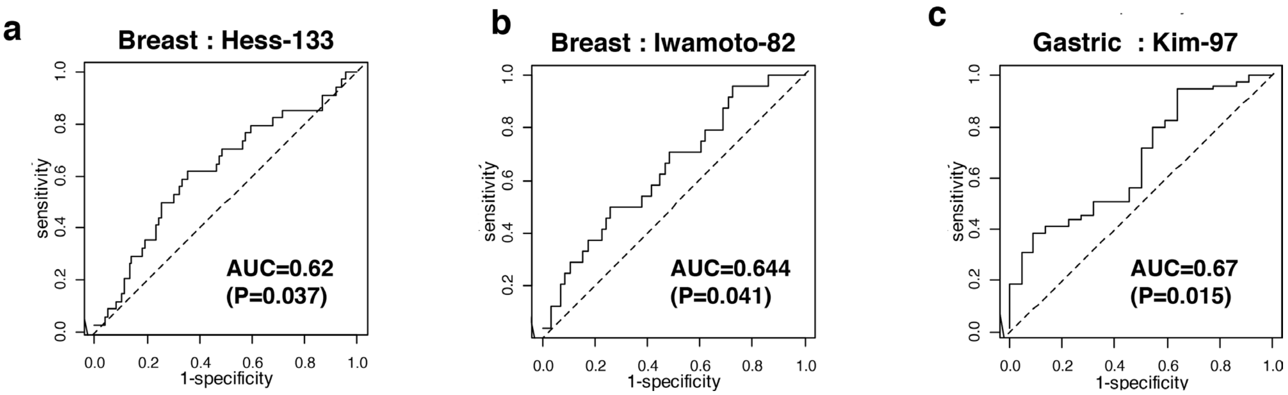

Supplementary Figure 6: 5-Fu: ROC curves of CONCORD prediction on breast and gastric cancers.

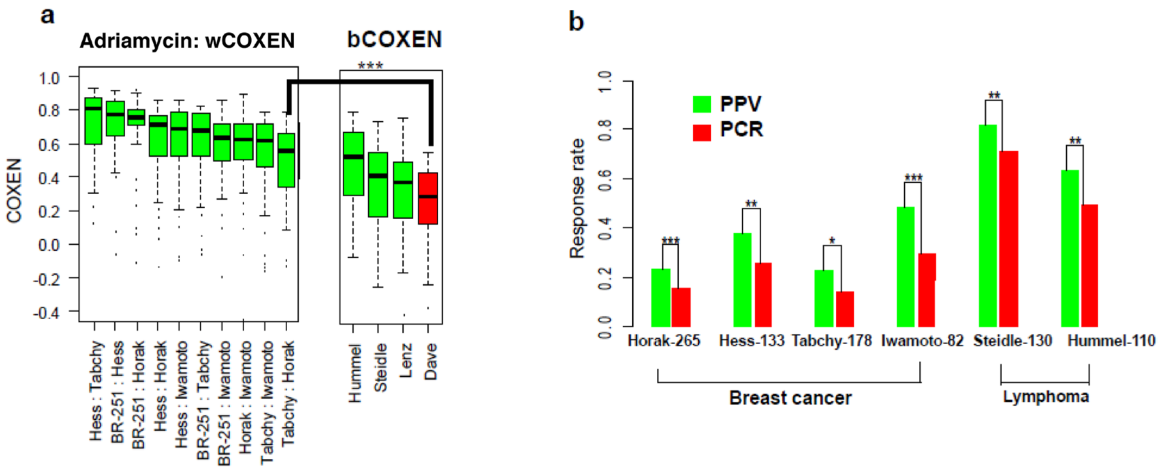

Supplementary Figure 7: Adriamycin: CONCORD prediction for breast cancer and lymphoma.

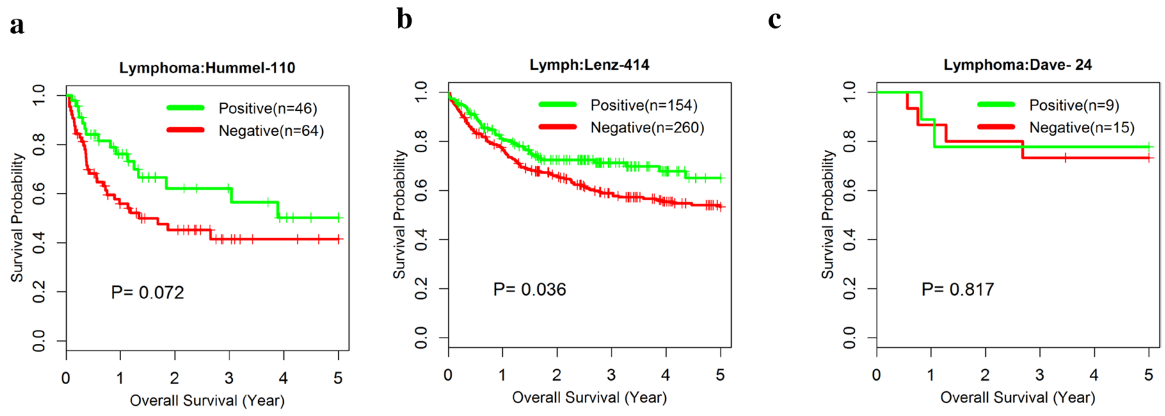

Supplementary Figure 8: Adriamycin: CONCORD prediction of overall survival in lymphoma.

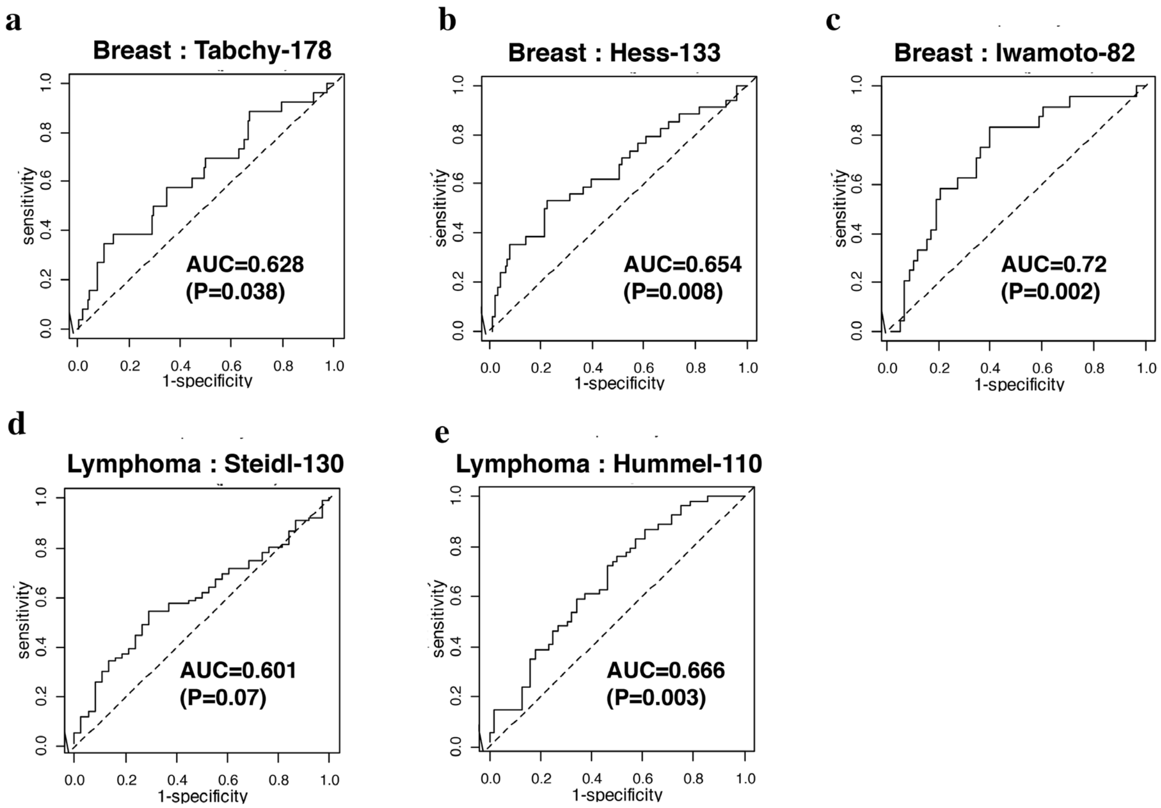

Supplementary Figure 9: Adriamycin: overall performance of CONCORD for breast and lymphoma.

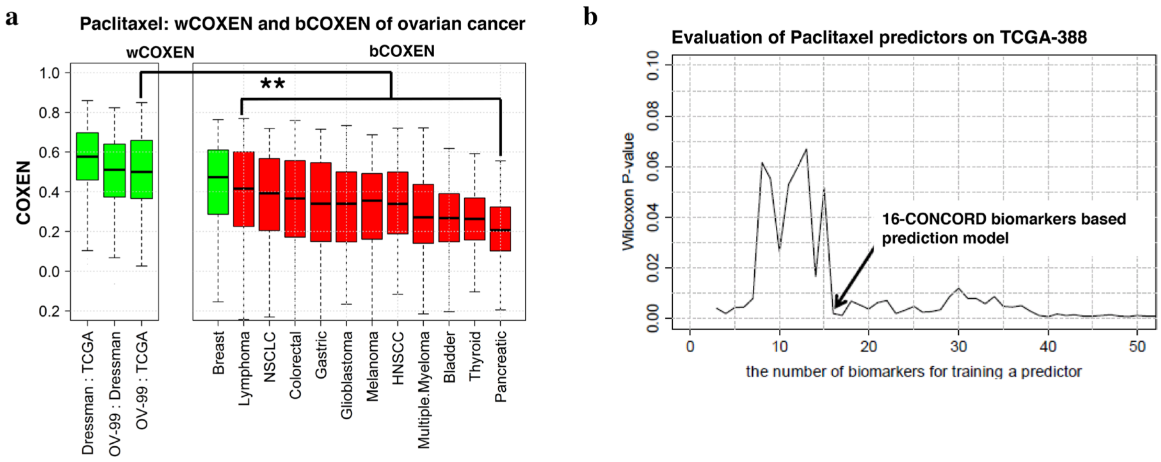

Supplementary Figure 10: Paclitaxel: CONCORD model development from ovarian cancer.

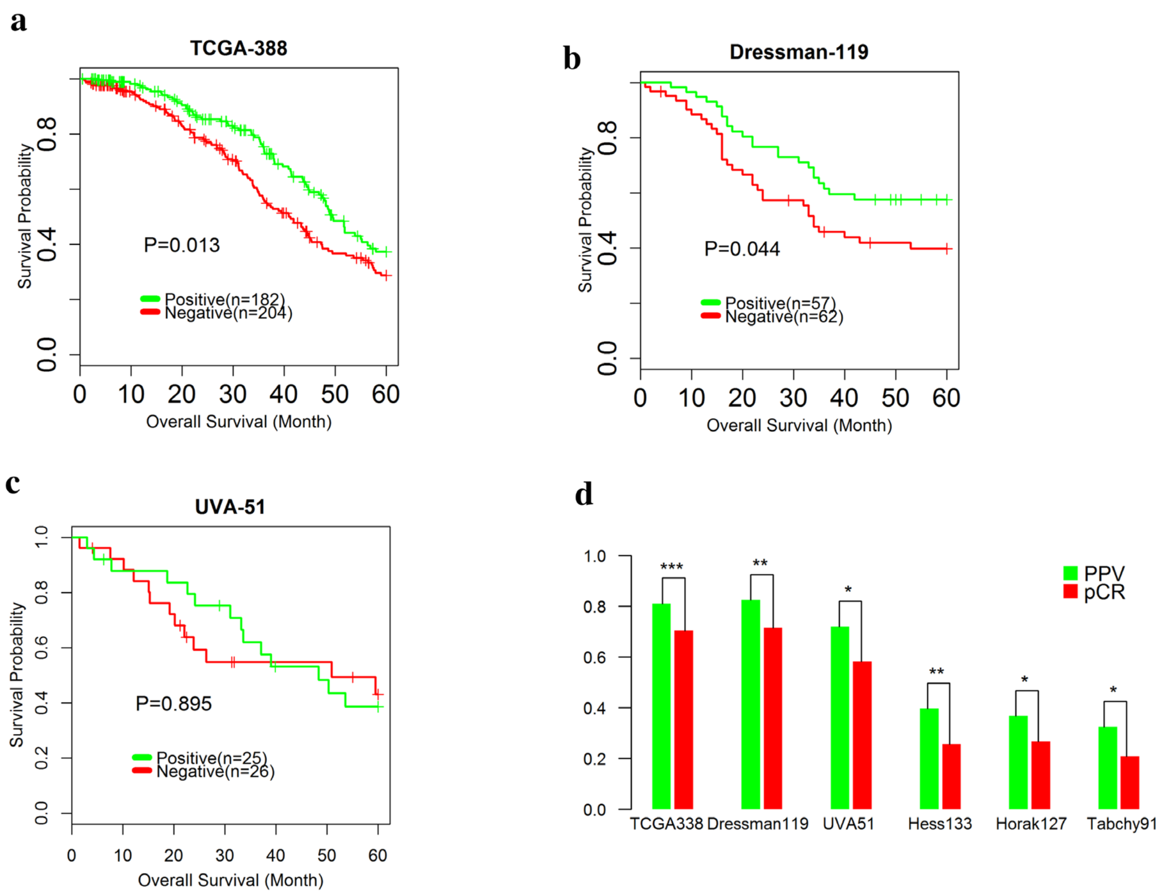

Supplementary Figure 11: Paclitaxel: Ovarian Cancer-to-Breast Cancer.

a

rank	cmap name	mean	n	enrichment	p	specificity	% non-null
107	paclitaxel	-0.304	6	-0.498	0.06786	0.1383	66

paclitaxel

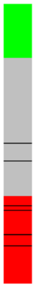

rank	batch	cmap name		dose	cell	score	up	down	instance_id
3037	613	paclitaxel		5 µM	HL60	0	-.313	-.202	2043
3434	714	paclitaxel		5 µM	PC3	0	-.387	-.179	6720
4398	633	paclitaxel		5 µM	MCF7	-.387	-.344	.157	1542
4506	611	paclitaxel		5 µM	PC3	-.411	-.246	.286	1959
5035	111	paclitaxel		100 nM	MCF7	-.493	-.345	.293	640
5272	729	paclitaxel		5 µM	MCF7	-.532	-.519	.170	5320

b

rank	cmap name	mean	n	enrichment	p	specificity	% non-null
73	doxorubicin	-0.634	3	-0.772	0.02430	0.2481	100

doxorubicin

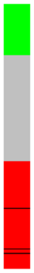

rank	batch	cmap name		dose	cell	score	up	down	instance_id
4707	708	doxorubicin		7 µM	MCF7	-.534	-.326	.179	5671
5635	655	doxorubicin		7 µM	MCF7	-.674	-.393	.244	3291
5754	712	doxorubicin		7 µM	PC3	-.695	-.449	.208	4610

Supplementary Figure 12: Connectivity Map Analysis of CONCORD Biomarkers.

Supplementary Table 1: The result of biomarker discovery, three-way COXEN analysis and model selection

See Supplementary File 1

Supplementary Table 2: Ingenuity Pathway Analysis of Paclitaxel biomarkers

See Supplementary File 2

Supplementary Table 3: Ingenuity Pathway Analysis of 5-FU biomarkers

See Supplementary File 3

Supplementary Table 4: Ingenuity Pathway Analysis of Adriamycin biomarkers

See Supplementary File 4

Supplementary Table 5: Complete list of biomarkers of CONCORD predictors

See Supplementary File 5

Supplementary Table 6: The list of gene expression and chemotherapeutic response data of cancer cell lines and cancer patient cohorts

See Supplementary File 6
