# Supplementary material for: CONCORD biomarker prediction for novel drug introduction to different cancer types
Source: Oncotarget. 2017 Dec 9;9(1):1091–106. doi: 10.18632/oncotarget.23124 (PMC5787421; doi:10.18632/oncotarget.23124)
Supplement: Supplementary file 2 [file oncotarget-09-1091-s002.docx]

**Supplementary Table** **1**. The result of biomarker discovery, three-way COXEN analysis and model selection

| Chemo/  targeted  therapy  agent | *the number of in vitro* drug sensitivity  biomarker (resource) | Three-way COXEN Analysis | | | Model  Selection  (Model test  cohort) | Threshold  of  Prediction  Score |
| --- | --- | --- | --- | --- | --- | --- |
|  |  | COXEN-set  (Cancer type: Patient Pattern set) | Pairwise Comparison of  Co-expression Pattern | CONCORD  Biomarkers |  |  |
| Paclitaxel | 202 (NCI-60)^$^  4 (GDSC)  2 (CCLE) | Breast: BR-251  Ovarian: OV-99 | NCI-60 vs Breast : 159 (78.7%)  NCI-60 vs Ovarian : 177 (87.6%)  Breast vs Ovarian : 184 (91.1%) | 142  (70.3%) | 16  (Hess-133) | 0.541 |
| 5-FU  (Fluoracil) | 611 (NCI-60) | Breast : BR-251  Gastric : GA-43 | NCI-60 vs Breast : 533 (87.2%)  NCI-60 vs Gastric : 545 (89.2%)  Breast vs Gastric : 560 (91.7%) | 475  (77.7%) | 7  (Tabchy-178) | 0.657 |
| Adriamycin | 499 (GDSC) ^$^  2 (NCI-60) | Breast: BR-251  Lymphoma: LY-111 | GDSC-648 vs Lymphoma:426 (85.4%)  GDSC-648 vs Breast: 444 (89%)  Breast vs Lymphoma: 450 (90.2%) | 377  (75.6%) | 56  (Horak-265) | 0.63 |
| Erlotinib | 96 (CCLE) ^$^  55 (GDSC)  83 (NCI-60) | NSCLC: EGFR-mut-49 |  |  |  |  |
| Vemurafenib | 125 (NCI-60) | Melanoma:VU-V600E |  |  |  |  |

^$^ These cancer cell line panels with the largest number of drug sensitivity biomarkers were considered used for following Three-way COXEN analysis and drug response prediction model development.
