# Supplementary material for: CONCORD biomarker prediction for novel drug introduction to different cancer types
Source: Oncotarget. 2017 Dec 9;9(1):1091–106. doi: 10.18632/oncotarget.23124 (PMC5787421; doi:10.18632/oncotarget.23124)
Supplement: Supplementary file 3 [file oncotarget-09-1091-s003.docx]

**Supplementary Table 2. Ingenuity Pathway Analysis of Paclitaxel biomarkers**

| **Top Networks** | | | |
| --- | --- | --- | --- |
| ID | **ID Associated Network Functions** | | score |
| 1 | Cell Cycle, DNA Replication, Recombination, and Repair, Cancer | | 56 |
| 2 | Cell-To-Cell Signaling and Interaction, Nervous System Development and Function, Tissue Development | | 42 |
| 3 | Cell Signaling, Developmental Disorder, Skeletal and Muscular Disorders | | 30 |
| 4 | Developmental Disorder, Organismal Injury and Abnormalities, Reproductive System Disease | | 27 |
| 5 | DNA Replication, Recombination, and Repair, Hematological Disease, Lipid Metabolism | | 26 |
| **Top Diseases and Bio Functions** | | | |
| **Disease and Disorders** | | | |
| ID | Name | p-value | # Molecules |
| 1 | Developmental Disorder | 2.50E-06 - 1.18E-02 | 29 |
| 2 | Cancer | 3.28E-05 - 1.18E-02 | 67 |
| 3 | Hematological Disease | 3.28E-05 - 1.18E-02 | 21 |
| 4 | Immunological Disease | 3.28E-05 - 1.18E-02 | 23 |
| 5 | Endocrine System Disorders | 2.26E-04 - 1.18E-02 | 15 |
| **Molecular and Cellular Functions** | | | |
| ID | Name | p-value | # Molecules |
| 1 | Cell Death and Survival | 2.69E-08 - 1.18E-02 | 50 |
| 2 | Cell Cycle | 1.24E-07 - 1.18E-02 | 30 |
| 3 | Cellular Development | 3.54E-07 - 1.18E-02 | 42 |
| 4 | Cellular Growth and Proliferation | 3.54E-07 - 1.18E-02 | 50 |
| 5 | Cell-To-Cell Signaling and Interaction | 3.50E-05 - 1.18E-02 | 13 |
| **Physiological System Development and Function** | | | |
| ID | Name | p-value | # Molecules |
| 1 | Connective Tissue Development and Function | 1.24E-07 - 1.18E-02 | 22 |
| 2 | Organismal Survival | 1.12E-06 - 5.41E-04 | 39 |
| 3 | Organismal Development | 1.27E-06 - 1.18E-02 | 37 |
| 4 | Hair and Skin Development and Function | 1.12E-05 - 1.18E-02 | 11 |
| 5 | Tissue Morphology | 2.11E-05 - 9.74E-03 | 31 |
| **Top Canonical Pathways** | | | |
| ID | Name | p-value | Ratio |
| 1 | Estrogen-mediated S-phase Entry | 3.77E-04 | 3/28 (0.107) |
| 2 | Granzyme B Signaling | 3.97E-03 | 2/18(0.111) |
| 3 | Cell Cycle: G1/S Checkpoint Regulation | 7.11E-03 | 3/72 (0.042) |
| 4 | Small Cell Lung Cancer Signaling | 8.70E-03 | 3/94 (0.032) |
| 5 | Tumoricidal Function of Hepatic Natural Killer Cells | 8.86E-03 | 2/27 (0.074) |
