# Supplementary material for: CONCORD biomarker prediction for novel drug introduction to different cancer types
Source: Oncotarget. 2017 Dec 9;9(1):1091–106. doi: 10.18632/oncotarget.23124 (PMC5787421; doi:10.18632/oncotarget.23124)
Supplement: Supplementary file 4 [file oncotarget-09-1091-s004.docx]

**Supplementary Table 3. Ingenuity Pathway Analysis of 5-FU biomarkers**

| Top Networks | | | |
| --- | --- | --- | --- |
| ID | Associated Network Functions | | Score |
| 1 | RNA Post-Transcriptional Modification, Amino Acid Metabolism, Small Molecule Biochemistry | | 50 |
| 2 | Amino Acid Metabolism, Small Molecule Biochemistry, DNA Replication, Recombination, and Repair | | 50 |
| 3 | Cell Morphology, Cellular Function and Maintenance, Cellular Assembly and Organization | | 48 |
| 4 | Cancer, Hematological Disease, Protein Synthesis | | 48 |
| 5 | Post-Translational Modification, Cell Morphology, Cell-To-Cell Signaling and Interaction | | 40 |
| Top Diseases and Bio Functions | | | |
|  | **Disease & Disorders** |  |  |
| ID | Name | p-value | # Molecules |
| 1 | Organismal Injury and Abnormalities | 4.39E-04 - 1.85E-02 | 85 |
| 2 | Cancer | 5.02E-04 - 1.85E-02 | 98 |
| 3 | Reproductive System Disease | 5.02E-04 - 1.85E-02 | 54 |
| 4 | Hematological Disease | 5.93E-04 - 1.85E-02 | 21 |
| 5 | Endocrine System Disorders | 1.01E-03 - 1.85E-02 | 6 |
|  | **Molecular and Cellular Functions** |  |  |
| ID | Name | p-value | # Molecules |
| 1 | Protein Synthesis | 2.54E-07 - 1.48E-02 | 50 |
| 2 | DNA Replication, Recombination, and Repair | 2.61E-07 - 1.85E-02 | 35 |
| 3 | Cellular Growth and Proliferation | 3.89E-06 - 1.85E-02 | 123 |
| 4 | RNA Post-Transcriptional Modification | 1.35E-05 - 1.85E-02 | 23 |
| 5 | Cellular Assembly and Organization | 2.47E-05 - 1.85E-02 | 73 |
|  | **Physiological System Development and Function** |  |  |
| ID | Name | p-value | # Molecules |
| 1 | Connective Tissue Development and Function | 8.61E-05 - 1.85E-02 | 35 |
| 2 | Organismal Survival | 8.72E-05 - 8.72E-05 | 84 |
| 3 | Nervous System Development and Function | 9.91E-05 - 1.85E-02 | 22 |
| 4 | Skeletal and Muscular System Development and Function | 1.75E-04 - 1.85E-02 | 16 |
| 5 | Embryonic Development | 1.01E-03 - 1.85E-02 | 23 |
| **Top Canonical Pathways** | | | |
| ID | Name | p-value | Ratio |
| 1 | EIF2 Signaling | 5.84E-08 | 17/201 (0.085) |
| 2 | Cell Cycle Control of Chromosomal Replication | 1.02E-05 | 6/34 (0.176) |
| 3 | Mitochondrial Dysfunction | 2.70E-04 | 11/215 (0.051) |
| 4 | Adenine and Adenosine Salvage I | 3.41E-04 2/9 | -0.222 |
| 5 | Glycine Biosynthesis I 3.41E-04 | 6-Feb | -0.333 |
