# Supplementary material for: CONCORD biomarker prediction for novel drug introduction to different cancer types
Source: Oncotarget. 2017 Dec 9;9(1):1091–106. doi: 10.18632/oncotarget.23124 (PMC5787421; doi:10.18632/oncotarget.23124)
Supplement: Supplementary file 5 [file oncotarget-09-1091-s005.docx]

**Supplementary Table 4. Ingenuity Pathway Analysis of Adriamycin biomarkers**

| **Top Networks** | | | |
| --- | --- | --- | --- |
| ID | Associated Network Functions | | Score |
| 1 | Cellular Function and Maintenance, Molecular Transport, Cancer | | 53 |
| 2 | RNA Post-Transcriptional Modification, Dermatological Diseases and Conditions, Hematological Disease | | 45 |
| 3 | Hereditary Disorder, Neurological Disease, Developmental Disorder | | 40 |
| 4 | Gene Expression, Lipid Metabolism, Molecular Transport | | 38 |
| 5 | Cell Signaling, Cell Morphology, Cellular Assembly and Organization | | 34 |
| **Top Diseases and Bio Functions** | | | |
| **Disease and Disorders** | | | |
| ID | **Name** | p-value | # Molecules |
| 1 | Neurological Disease | 1.51E-06 - 1.56E-02 | 75 |
| 2 | Psychological Disorders | 2.57E-05 - 2.34E-03 | 41 |
| 3 | Hereditary Disorder | 5.09E-05 - 1.56E-02 | 53 |
| 4 | Skeletal and Muscular Disorders | 5.46E-05 - 1.56E-02 | 43 |
| 5 | Cardiovascular Disease | 2.41E-04 - 1.56E-02 | 11 |
| **Molecular and Cellular Functions** | | | |
| ID | **Name** | p-value | # Molecules |
| 1 | Molecular Transport | 5.79E-07 - 1.56E-02 | 75 |
| 2 | RNA Post-Transcriptional Modification | 5.30E-06 - 1.56E-02 | 21 |
| 3 | Lipid Metabolism | 1.12E-05 - 1.56E-02 | 32 |
| 4 | Small Molecule Biochemistry | 1.12E-05 - 1.56E-02 | 41 |
| 5 | Cell Death and Survival | 5.09E-05 - 1.56E-02 | 101 |
| **Physiological System Development and Function** | | | |
| ID | **Name** | p-value | # Molecules |
| 1 | Endocrine System Development and Function | 1.12E-05 - 1.56E-02 | 16 |
| 2 | Embryonic Development | 3.06E-05 - 1.56E-02 | 39 |
| 3 | Organismal Survival | 7.41E-05 - 8.66E-05 | 74 |
| 4 | Behavior | 1.22E-04 - 1.21E-02 | 37 |
| 5 | Connective Tissue Development and Function | 1.97E-04 - 1.56E-02 | 18 |
| **Top Canonical Pathways** | | | |
| ID | Name | p-value | Ratio |
| 1 | Role of p14/p19ARF in Tumor Suppression | 1.15E-03 | 4/35 (0.114) |
| 2 | MIF Regulation of Innate Immunity | 3.7E-03 4/52 | -0.077 |
| 3 | Role of Oct4 in Mammalian Embryonic Stem Cell Pluripotency | 5.19E-03 | 4/52 (0.077) |
| 4 | Maturity Onset Diabetes of Young (MODY) Signaling | 5.25E-03 | 3/33 (0.091) |
| 5 | Wnt/b-catenin Signaling | 5.42E-03 | 8/175 (0.046) |
