# Supplementary material for: CONCORD biomarker prediction for novel drug introduction to different cancer types
Source: Oncotarget. 2017 Dec 9;9(1):1091–106. doi: 10.18632/oncotarget.23124 (PMC5787421; doi:10.18632/oncotarget.23124)
Supplement: Supplementary file 6 [file oncotarget-09-1091-s006.docx]

**Supplementary Table 5.** . **Complete list of biomarkers of CONCORD predictors**

| **Paclitaxel Predictor** | | | | | | | | | | | | |
| --- | --- | --- | --- | --- | --- | --- | --- | --- | --- | --- | --- | --- |
| **id** | | **probeset** | | **Association statistics** | **p-value** | | | **q-value** | | **Symbol** | | **Entrez Gene Name** |
| 1 | | 202200_s_at | | 5.525 | 3.30E-08 | | | 6.07E-04 | | SRPK1 | | SRSF protein kinase 1 |
| 2 | | 204881_s_at | | -5.201 | 1.98E-07 | | | 1.22E-03 | | UGCG | | UDP-glucose ceramide glucosyltransferase |
| 3 | | 209278_s_at | | -5.199 | 2.00E-07 | | | 1.22E-03 | | TFPI2 | | tissue factor pathway inhibitor 2 |
| 4 | | 219733_s_at | | 5.074 | 3.90E-07 | | | 1.71E-03 | | SLC27A5 | | solute carrier family 27 (fatty acid transporter), member 5 |
| 5 | | 214247_s_at | | -4.998 | 5.80E-07 | | | 1.71E-03 | | DKK3 | | dickkopf 3 homolog (Xenopus laevis) |
| 6 | | 202305_s_at | | -4.990 | 6.02E-07 | | | 1.71E-03 | | FEZ2 | | fasciculation and elongation protein zeta 2 (zygin II) |
| 7 | | 202431_s_at | | 4.859 | 1.18E-06 | | | 2.71E-03 | | MYC | | v-myc myelocytomatosis viral oncogene homolog (avian) |
| 8 | | 201537_s_at | | -4.782 | 1.74E-06 | | | 2.71E-03 | | DUSP3 | | dual specificity phosphatase 3 |
| 9 | | 201474_s_at | | -4.778 | 1.77E-06 | | | 2.71E-03 | | ITGA3 | | integrin, alpha 3 (antigen CD49C, alpha 3 subunit of VLA-3 receptor) |
| 10 | | 211458_s_at | | -4.759 | 1.94E-06 | | | 2.74E-03 | | GABARAPL3 | | GABA(A) receptors associated protein like 3, pseudogene |
| 11 | | 219687_at | | -4.668 | 3.04E-06 | | | 3.73E-03 | | HHAT | | hedgehog acyltransferase |
| 12 | | 204794_at | | 4.596 | 4.30E-06 | | | 4.65E-03 | | DUSP2 | | dual specificity phosphatase 2 |
| 13 | | 211566_x_at | | -4.494 | 6.99E-06 | | | 6.11E-03 | | BRE | | brain and reproductive organ-expressed (TNFRSF1A modulator) |
| 14 | | 205453_at | | -4.413 | 1.02E-05 | | | 7.79E-03 | | HOXB2 | | homeobox B2 |
| 15 | | 201401_s_at | | 4.378 | 1.20E-05 | | | 8.80E-03 | | ADRBK1 | | adrenergic, beta, receptor kinase 1 |
| 16 | | 218979_at | | 4.336 | 1.45E-05 | | | 9.63E-03 | | RMI1 | | RMI1, RecQ mediated genome instability 1, homolog (S. cerevisiae) |
| **5-FU Predictor** | | | | | | | | | | | | |
| **id** | | **probeset** | | **Drug sensitivity**  **Association statistics** | **P-value** | | | **q-value** | | **Gene**  **Symbol** | | **Entrez Gene Name** |
| 1 | | 205129_at | | -0.595 | 5.21E-07 | | | 0.009569 | | NPM3 | | nucleophosmin/nucleoplasmin 3 |
| 2 | | 201391_at | | -0.568 | 2.22E-06 | | | 0.020404 | | TRAP1 | | TNF receptor-associated protein 1 |
| 3 | | 203785_s_at | | -0.536 | 1.04E-05 | | | 0.037258 | | DDX28 | | DEAD (Asp-Glu-Ala-Asp) box polypeptide 28 |
| 4 | | 218670_at | | -0.530 | 1.32E-05 | | | 0.037258 | | PUS1 | | pseudouridylate synthase 1 |
| 5 | | 205895_s_at | | -0.530 | 1.35E-05 | | | 0.037258 | | NOLC1 | | nucleolar and coiled-body phosphoprotein 1 |
| 6 | | 212656_at | | -0.525 | 1.66E-05 | | | 0.038019 | | TSFM | | Ts translation elongation factor, mitochondrial |
| 7 | | 218001_at | | -0.513 | 2.76E-05 | | | 0.043376 | | MRPS2 | | mitochondrial ribosomal protein S2 |
| **Adriamycin Predictor** | | | | | | | | | | | | |
| **id** | **probeset** | | **Association statistics** | | | **p-value** | **q-value** | | **Symbol** | | **Entrez Gene Name** | |
| 1 | 204524_at | | 0.2216 | | | 0.0000 | 0.0005 | | PDPK1 | | 3-phosphoinositide dependent protein kinase-1 | |
| 2 | 211916_s_at | | 0.2201 | | | 0.0000 | 0.0005 | | MYO1A | | myosin IA | |
| 3 | 204623_at | | 0.2188 | | | 0.0000 | 0.0005 | | TFF3 | | trefoil factor 3 (intestinal) | |
| 4 | 221567_at | | 0.2105 | | | 0.0000 | 0.0009 | | NOL3 | | nucleolar protein 3 (apoptosis repressor with CARD domain) | |
| 5 | 59625_at | | 0.2091 | | | 0.0000 | 0.0009 | | NOL3 | | nucleolar protein 3 (apoptosis repressor with CARD domain) | |
| 6 | 209222_s_at | | 0.2065 | | | 0.0000 | 0.0010 | | OSBPL2 | | oxysterol binding protein-like 2 | |
| 7 | 222103_at | | -0.2015 | | | 0.0000 | 0.0015 | | ATF1 | | activating transcription factor 1 | |
| 8 | 215543_s_at | | 0.2014 | | | 0.0000 | 0.0015 | | LARGE | | like-glycosyltransferase | |
| 9 | 213200_at | | 0.2001 | | | 0.0000 | 0.0015 | | SYP | | synaptophysin | |
| 10 | 204744_s_at | | -0.2001 | | | 0.0000 | 0.0015 | | IARS | | isoleucyl-tRNA synthetase | |
| 11 | 218889_at | | -0.1975 | | | 0.0000 | 0.0017 | | NOC3L | | nucleolar complex associated 3 homolog (S. cerevisiae) | |
| 12 | 202972_s_at | | 0.1965 | | | 0.0000 | 0.0017 | | FAM13A | | family with sequence similarity 13, member A | |
| 13 | 205093_at | | 0.1963 | | | 0.0000 | 0.0017 | | PLEKHA6 | | pleckstrin homology domain containing, family A member 6 | |
| 14 | 216930_at | | 0.1958 | | | 0.0000 | 0.0017 | | HNF1A | | HNF1 homeobox A | |
| 15 | 218595_s_at | | -0.1956 | | | 0.0000 | 0.0017 | | HEATR1 | | HEAT repeat containing 1 | |
| 16 | 221879_at | | 0.1948 | | | 0.0000 | 0.0018 | | CALML4 | | calmodulin-like 4 | |
| 17 | 214495_at | | 0.1937 | | | 0.0000 | 0.0019 | | CACNG2 | | calcium channel, voltage-dependent, gamma subunit 2 | |
| 18 | 221566_s_at | | 0.1932 | | | 0.0000 | 0.0019 | | NOL3 | | nucleolar protein 3 (apoptosis repressor with CARD domain) | |
| 19 | 212858_at | | 0.1923 | | | 0.0000 | 0.0020 | | PAQR4 | | progestin and adipoQ receptor family member IV | |
| 20 | 208234_x_at | | 0.1917 | | | 0.0000 | 0.0020 | | FGFR2 | | fibroblast growth factor receptor 2 | |
| 21 | 209993_at | | 0.1913 | | | 0.0000 | 0.0020 | | ABCB1 | | ATP-binding cassette, sub-family B (MDR/TAP), member 1 | |
| 22 | 213577_at | | 0.1910 | | | 0.0000 | 0.0020 | | SQLE | | squalene epoxidase | |
| 23 | 211184_s_at | | 0.1910 | | | 0.0000 | 0.0020 | | USH1C | | Usher syndrome 1C (autosomal recessive, severe) | |
| 24 | 206469_x_at | | 0.1899 | | | 0.0000 | 0.0022 | | AKR7A3 | | aldo-keto reductase family 7, member A3 (aflatoxin aldehyde reductase) | |
| 25 | 208063_s_at | | 0.1896 | | | 0.0000 | 0.0022 | | CAPN9 | | calpain 9 | |
| 26 | 205137_x_at | | 0.1891 | | | 0.0000 | 0.0023 | | USH1C | | Usher syndrome 1C (autosomal recessive, severe) | |
| 27 | 221992_at | | 0.1886 | | | 0.0000 | 0.0023 | | NPIPL2 | | nuclear pore complex interacting protein-like 2 | |
| 28 | 210390_s_at | | 0.1869 | | | 0.0000 | 0.0025 | | CCL15 | | chemokine (C-C motif) ligand 15 | |
| 29 | 201964_at | | -0.1868 | | | 0.0000 | 0.0025 | | SETX | | senataxin | |
| 30 | 201726_at | | -0.1867 | | | 0.0000 | 0.0025 | | ELAVL1 | | ELAV (embryonic lethal, abnormal vision, Drosophila)-like 1 (Hu antigen R) | |
| 31 | 209233_at | | -0.1862 | | | 0.0000 | 0.0026 | | EMG1 | | EMG1 nucleolar protein homolog (S. cerevisiae) | |
| 32 | 218594_at | | -0.1854 | | | 0.0000 | 0.0027 | | HEATR1 | | HEAT repeat containing 1 | |
| 33 | 211401_s_at | | 0.1849 | | | 0.0000 | 0.0028 | | FGFR2 | | fibroblast growth factor receptor 2 | |
| 34 | 205019_s_at | | 0.1842 | | | 0.0000 | 0.0029 | | VIPR1 | | vasoactive intestinal peptide receptor 1 | |
| 35 | 214898_x_at | | 0.1830 | | | 0.0000 | 0.0033 | | MUC3B | | mucin 3B, cell surface associated | |
| 36 | 200014_s_at | | -0.1826 | | | 0.0000 | 0.0033 | | HNRNPC | | heterogeneous nuclear ribonucleoprotein C (C1/C2) | |
| 37 | 210264_at | | 0.1823 | | | 0.0000 | 0.0033 | | GPR35 | | G protein-coupled receptor 35 | |
| 38 | 220354_at | | 0.1810 | | | 0.0000 | 0.0036 | | MCF2L-AS1 | | MCF2L antisense RNA 1 | |
| 39 | 206374_at | | 0.1810 | | | 0.0000 | 0.0036 | | DUSP8 | | dual specificity phosphatase 8 | |
| 40 | 213397_x_at | | 0.1809 | | | 0.0000 | 0.0036 | | RNASE4 | | ribonuclease, RNase A family, 4 | |
| 41 | 203421_at | | 0.1809 | | | 0.0000 | 0.0036 | | TP53I11 | | tumor protein p53 inducible protein 11 | |
| 42 | 208477_at | | 0.1801 | | | 0.0000 | 0.0038 | | KCNC1 | | potassium voltage-gated channel, Shaw-related subfamily, member 1 | |
| 43 | 216381_x_at | | 0.1799 | | | 0.0000 | 0.0038 | | AKR7A3 | | aldo-keto reductase family 7, member A3 (aflatoxin aldehyde reductase) | |
| 44 | 209173_at | | 0.1796 | | | 0.0000 | 0.0039 | | AGR2 | | anterior gradient 2 homolog (Xenopus laevis) | |
| 45 | 215066_at | | 0.1787 | | | 0.0000 | 0.0042 | | PTPRF | | protein tyrosine phosphatase, receptor type, F | |
| 46 | 200954_at | | 0.1786 | | | 0.0000 | 0.0042 | | ATP6V0C | | ATPase, H+ transporting, lysosomal 16kDa, V0 subunit c | |
| 47 | 201030_x_at | | -0.1774 | | | 0.0000 | 0.0045 | | LDHB | | lactate dehydrogenase B | |
| 48 | 208107_s_at | | 0.1769 | | | 0.0000 | 0.0046 | | LOC81691 | | exonuclease NEF-sp | |
| 49 | 209847_at | | 0.1768 | | | 0.0000 | 0.0046 | | CDH17 | | cadherin 17, LI cadherin (liver-intestine) | |
| 50 | 210611_s_at | | 0.1767 | | | 0.0000 | 0.0046 | | DTNA | | dystrobrevin, alpha | |
| 51 | 221769_at | | 0.1752 | | | 0.0000 | 0.0053 | | SPSB3 | | splA/ryanodine receptor domain and SOCS box containing 3 | |
| 52 | 205795_at | | 0.1745 | | | 0.0000 | 0.0057 | | NRXN3 | | neurexin 3 | |
| 53 | 215624_at | | 0.1740 | | | 0.0000 | 0.0058 | | TSC2 | | tuberous sclerosis 2 | |
| 54 | 219562_at | | 0.1738 | | | 0.0000 | 0.0058 | | RAB26 | | RAB26, member RAS oncogene family | |
| 55 | 200899_s_at | | -0.1738 | | | 0.0000 | 0.0058 | | MGEA5 | | meningioma expressed antigen 5 (hyaluronidase) | |
| 56 | 205141_at | | 0.1734 | | | 0.0000 | 0.0060 | | ANG | | angiogenin, ribonuclease, RNase A family, 5 | |
