# Supplementary material for: CONCORD biomarker prediction for novel drug introduction to different cancer types
Source: Oncotarget. 2017 Dec 9;9(1):1091–106. doi: 10.18632/oncotarget.23124 (PMC5787421; doi:10.18632/oncotarget.23124)
Supplement: Supplementary file 7 [file oncotarget-09-1091-s007.docx]

**Supplementary Table 6.** The list of gene expression and chemotherapeutic response data of cancer cell lines and cancer patient cohorts

| **Cancer**  **type** | **Name** | **Array**  **platform** | **GEO access ID**  **or website** | **Drug**  **response** | **Number of patients**  **(chemotheraypy**  **outcome)** | | **Chemo**  **therapy**  **/ drugs** | **Role of data** |
| --- | --- | --- | --- | --- | --- | --- | --- | --- |
| Cell line | NCI-60 | HG-U133A |  | GI-50 |  | | > 250,000 compounds | Biomarker Discovery |
|  | GDSC-648 | HT-U133A |  | IC-50 |  | | 138 drugs | Biomarker Discovery |
|  | CCLE-504 | HG-U133+2 |  | IC-50 |  | | 24 drugs | Biomarker Discovery |
| Breast  Cancer | BR-251 | HG-U133A | GSE3494 |  |  | | - | COXEN set |
|  | Horak-297 | HG-U133+2 | GSE41998 | pCR |  | | AC + Taxol or  AC+Ixabepilone | Model selection |
|  | Hess-133 | HG-U133A | GSE20194 | PFS, pCR | 34pCR / 99 RD | | TFAC | Model selectin /  Self prediction |
|  | Tabchy-178 | HG-U133A | GSE20271 | pCR |  | | TFAC | Self prediction |
|  | Iwamoto-82 | HG-U133A | GSE23988 | pCR |  | | FAC/FEC | Self prediction |
| Ovarian  Cancer | OV-99 | HG-U133A | GSE6008 |  |  | | - | COXEN set |
|  | Dressman-119 | HG-U133A | http://Cagrid1.duhs.duke.  edu:38080/caarray | pCR, OS | 85 R / 34 NR | | Taxol + Carboplatin | Cross-prediction |
|  | UVA-51 | HG-U133A | GSE30161 | pCR, PFS, OS | 28 R / 23 NR | | Taxol + Carboplatin | Cross-prediction |
|  | TCGA-388 | HG-U133A | TCGA | pCR, PFS, OS | 272 CR, 54 PR, 25 SD, 36 PD | | Taxol+Cisplatin | Cross-prediction |
| Lymphoma | LY-111 | HG-U133A | GSE4475 |  |  | - | | COXEN set |
|  | Steidl-130 | HG-U133+2 | GSE17920 | relapse type  (early / late) | 92 No, 19 late, 9 early relapse, 10 refractory | ABVD | | Cross-prediction |
|  | Lenz-414 | HG-U133+2 | GSE10846 | OS |  | R-CHOP | | Cross-prediction |
|  | Hummel-110 | HG-U133A | GSE4475 | pCR, OS | 72 CR, 15 PR, 2 NC, 21 PD | R-CHOP | | Cross-prediction |
|  | Dave-24 | HG-U133+2 | GSE17372  http://Limpp.nih.gov/BL | OS |  | CHOP | | Cross-prediction |
| Gastric | GA-43 | HG-U133A | GSE22377 |  |  |  | | COXEN set |
|  | Kim-96 | HG-U133+2 | GSE14208 | pCR | 74 / 22 | 5-Fu + Cisplatin | | Cross-prediction |
| Non Small Cell Lung | NCI-443 | HG-U133A | NCI Caarray  Access id : 182 |  | 433 ADC (150 stage I, 251 stage II, 28 stage III, 12 stage IV) |  | | wCOXEN |
|  | Zhu-90 | HG-U133A | GSE14814 |  | 28 ADC, 10 LCUC, 52 SQCC(45 stage I and 45 stage II primary tumor) |  | | COXEN-set for bCOXEN |
|  | Kuner-58 | HG-U133+2 | GSE10245 |  | 40 ADC, 18 SQCC |  | | wCOXEN |
|  | Tiwan-55 | HG-U133+2 | GSE19804 |  | 56 ADC, 3 BCC, 1 SQCC (35 stage I, 12 stage II, 12 stage III, 1 stage IV) |  | | wCOXEN |
|  | IGR-103 | HG-U133+2 | E-MTAB-923 |  | 103 ADC (49 with mutant EGFR and 54 with wildtype EGFR) |  | | COXEN-set  for Erlotinib |
|  |  |  |  |  |  |  |  |  |
| Melanoma | DFCI-31 | HG-U133A | GSE46517 |  | Primary Melanoma |  | | COXEN-set for bCOXEN |
|  | VU-58 | HG-U133A | GSE15605 |  | 22 BRAF V600E and 36 non-V600E |  | | COXEN-set for  Vemurafenib |
|  | Duke-52 | HG-U133+2 | GSE19293 |  | 19 BRAF mutation and  22 BRAF wild type |  | | wCOXEN |
| Bladder | Carbayo-89 | HG-U133A | TABM-147* |  |  | - | | COXEN set |
| Colorectal | Jorissen-172 | HG-U133A | GSE14333 |  |  |  | | COXEN-set |
| Head& Neck | Pavon-68 | HG-U133A | GSE 23036 |  |  |  | | COXEN-set |
| Multiple Myeoloma | Mulligan-264 | HG-U133+2 | GSE39754 |  |  |  | | COXEN-set |
| Pancreatic | Badea-36 | HG-U133+2 | GSE15471 |  |  |  | | COXEN-set |
| Thyroid | Giordano-95 | HG-U133A | GSE27155 |  |  |  | | COXEN-set |

**pCR** : pathologic complete response, **OS** : overall survival, **PFS** : progression free survival
**GI-50** : 50% growth inhibition, **IC-50** : the half maximal inhibitory concentration, **NCI** : National Cancer Institute
**ADC**: Adenocarcinoma, **BCC**: Bronchioloaveolar carcinoma, **LCUC**: Large-cell undifferenciated carcinoma, **SQCC** : Squamous cell carcinoma
**^*^** : Access ID of gene expression data at ArrayExpress
